# Stability and Test–Retest Reliability of the Individual Acoustic Reference for Within-Subject Analysis of Hearing Device-Processed Speech: The SpettroREM Method

**DOI:** 10.3390/audiolres16050158

**Published:** 2026-10-11

**Authors:** Sandro Burdo, Edoardo Carini, Giuseppe Impalà, Davide Brotto

**Affiliations:** 1 Independent Researcher, 21100 Varese, Italy; 2 Institute for Maternal and Child Health–IRCCS Burlo Garofolo, 34137 Trieste, Italy; edoardo.carini@burlo.trieste.it; 3 UOC Otorinolaringoiatria/Otorhinolaryngology–Head and Neck Section, Azienda Ospedale-Università Padova, Via Giustiniani 2, 35128 Padova, Italy; giuseppe.impala@aopd.veneto.it (G.I.); davide.brotto@unipd.it (D.B.); 4 Department of Neurosciences, University of Padova, 35122 Padova, Italy

**Keywords:** real-ear measurement, hearing aids, speech acoustics, test–retest reliability, Praat, within-subject design

## Abstract

**Objective:** Modern hearing devices modify speech beyond simple amplification, whereas Real-Ear Measurement (REM) primarily quantifies the sound level delivered to the ear as a function of frequency. SpettroREM was developed to characterize spectral and temporal signal structure by comparing each aided recording with the corresponding unaided recording, termed the Individual Acoustic Reference (IAR). This study evaluates the stability and test–retest reliability of the unaided reference and characterizes the external recording pathway; it does not assess aided–unaided differences. **Design:** The descriptor framework was developed from preliminary auditory and visual analysis of four unaided ear-canal recordings. Established acoustic measures available in Praat were progressively extended to predefined speech segments, local measurements, derived spectral and temporal measures, vowel geometry, envelope dynamics, and speech contrast. The resulting exploratory library comprised 44 measures; 37 were designated as the principal descriptor set and organized into six complementary domains, whereas seven derived or partially overlapping measures were retained as exploratory outputs. REM recordings acquired using AURICAL FreeFit (Natus) were processed through a semi-automated Praat workflow. Descriptor variability was characterized using descriptive distributional measures and, where appropriate, coefficients of variation. Test–retest reliability was assessed using ICC(A,1) with 95% participant-level bootstrap confidence intervals, together with mean absolute differences, Bland–Altman limits of agreement, and repeatability coefficients. The external recording pathway was assessed through electrical loopback and acoustic comparison tests. **Study Sample:** This study included two complementary datasets: 26 unaided ear recordings for baseline descriptor characterization and bilateral test–retest recordings from 25 participants. The primary reliability analysis included 24 right ears because one right ear was excluded owing to tympanic membrane perforation. Test–retest recordings from all 25 contralateral left ears were analyzed separately as a within-sample sensitivity analysis to assess the robustness of the principal reliability pattern to the choice of ear used for the primary analysis. Right- and left-ear observations were not pooled. **Results:** Global spectral descriptors showed limited variability and the most consistent test–retest agreement in the primary right-ear analysis. In the exploratory post hoc tier classification, the seven Core descriptors showed ICC(A,1) values ranging from 0.906 to 0.984, with the lower bound of the 95% confidence interval remaining ≥0.75 for all Core descriptors. Mean absolute test–retest differences for the LTASS bands were 0.31 dB at 0–1 kHz, 0.50 dB at 1–4 kHz, and 0.88 dB at 4–8 kHz. The contralateral sensitivity analysis reproduced high repeatability of the three LTASS band levels, with ICCs ranging from 0.835 to 0.940, although it did not constitute an independent replication. Repeatability was less consistent for several recording-level-dependent, segmental, vowel-derived, and temporal-envelope descriptors, some of which were occasionally non-evaluable because of insufficient signal energy. Electrical testing showed no relevant spectral alteration or clipping in the cable–audio interface–Praat segment under the tested settings. External-recording repeatability was high across the primary mono-cable validation and the secondary stereo bridge experiment (*r* = 0.976–0.998), but frequency-dependent differences were observed between the OTOsuite display and the simultaneous external recording. The two acquisition outputs should therefore not be considered numerically interchangeable. **Conclusions:** Under standardized acquisition conditions, selected properties of the unaided IAR, particularly its global spectral organization, can be measured with good test–retest repeatability. Technical assessment supports internally consistent measurements using the tested AURICAL FreeFit–mono TS cable–external audio interface–Praat pathway but does not establish absolute spectral accuracy or numerical equivalence across acquisition outputs or platforms. The Core, Candidate, and Secondary classification is exploratory, post hoc, and requires confirmation in an independent sample. This study provides a preliminary reliability assessment of the unaided reference and its development-derived 37-descriptor library; it does not validate the complete SpettroREM method. Direct aided–unaided studies are required to establish sensitivity to device-induced transformations, aided-recording repeatability, clinical and perceptual relevance, and the most informative non-redundant descriptor subset.

## 1. Introduction

Probe-microphone Real-Ear Measurement (REM) is widely regarded as the clinical standard for verifying hearing-aid fittings. By measuring sound pressure levels within the external auditory canal, REM enables clinicians to determine whether the hearing device’s output matches prescribed amplification targets. REM remains an essential component of evidence-based hearing rehabilitation [1,2,3]. Recent evidence confirms the clinical relevance of probe-microphone verification and shows that its incremental effect on target matching may vary by hearing loss, fitting software, frequency, and initial-fit accuracy [4]. Conventional REM was developed primarily to evaluate frequency-dependent amplification and audibility, for which real-ear output level provides the principal measure of fitting performance.

Contemporary hearing devices no longer operate solely as amplifiers. Multichannel compression, adaptive directionality, feedback cancellation, frequency lowering, noise reduction, and increasingly artificial intelligence-based processing can modify the incoming speech signal dynamically [5,6,7]. Consequently, devices producing similar real-ear output levels may nevertheless differ in the spectral and temporal organization of their output. These differences may involve long-term spectral distribution, vowel formant structure, fricative energy, intensity contrast, and temporal-envelope characteristics, which contribute to the acoustic representation of speech [8]. Although contemporary REM systems frequently use running speech as the test signal, their principal outputs remain level- and spectrum-based measures. They therefore describe the amount and frequency distribution of the acoustic energy reaching the probe-microphone measurement point but provide limited information about how hearing-device processing modifies the segmental and temporal structure of speech.

Several objective methods have been proposed to quantify processing-related speech modifications, including intelligibility- and quality-prediction models such as STOI [9] and HASPI/HASQI [7,10,11], coherence-based measures [12], and the phase-inversion method described by Hagerman and Olofsson [13]. These methods address important but partly different objectives and, depending on the method, require controlled reference signals, paired clean and processed recordings, specific presentation conditions, or model assumptions. They were not developed primarily to provide a multidimensional description of the speech signal recorded directly within an individual ear canal during routine REM.

**Figure 1:**
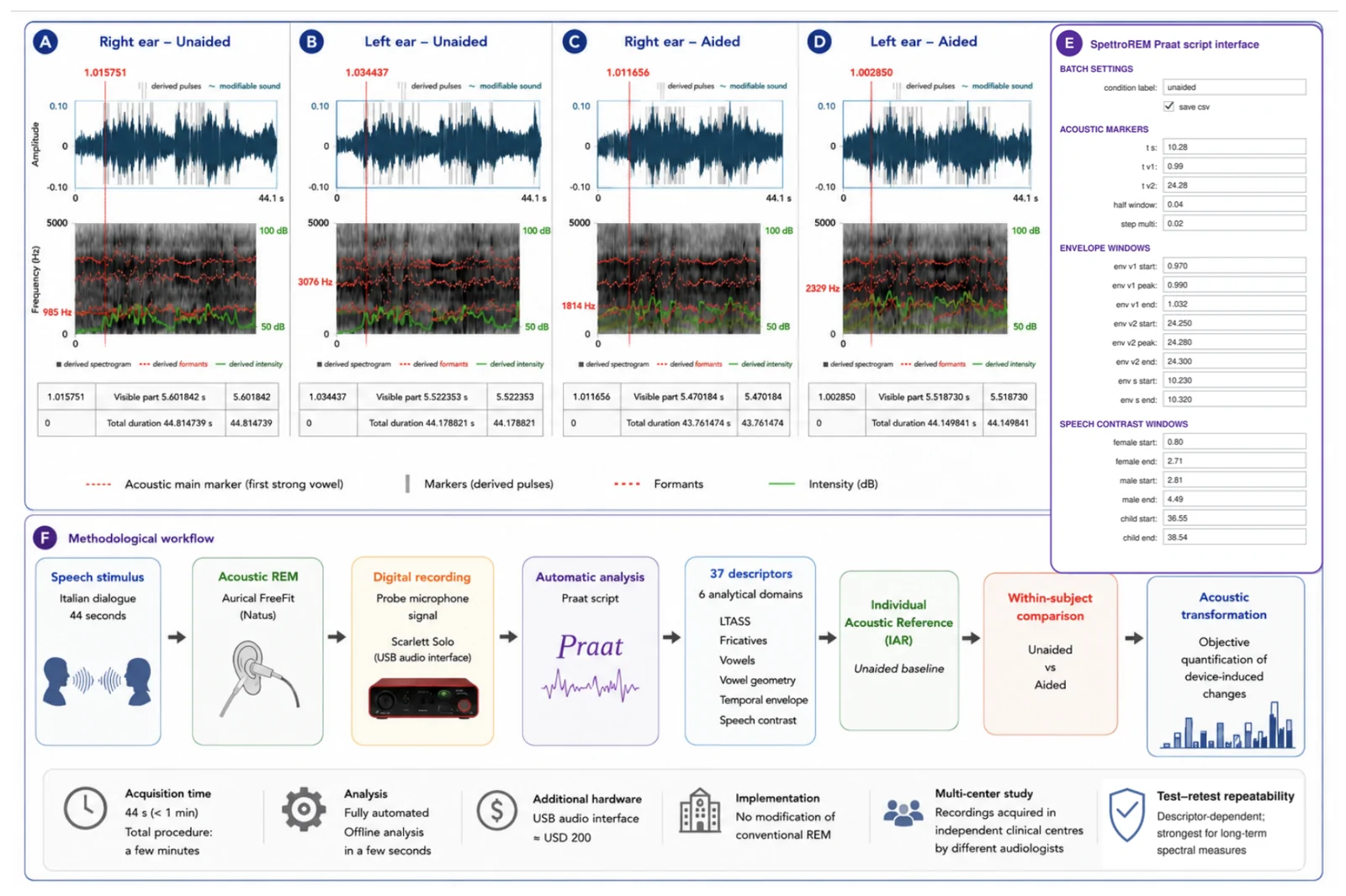
Overview of the SpettroREM Method. Representative real-ear recordings from the right and left ears under unaided and aided conditions (panels (**A**–**D**)) are shown alongside the dedicated Praat script interface (panel (**E**)) and the methodological workflow (panel (**F**)). Following temporal alignment, the SpettroREM script automatically processes the recordings and extracts 37 acoustic descriptors organized into six complementary analytical domains. The resulting Individual Acoustic Reference, unaided, serves as the baseline for paired within-subject comparisons of hearing device-induced acoustic transformations.

The SpettroREM method was developed to complement conventional REM by providing a descriptive, multidimensional acoustic analysis of the intracanal speech signal. Rather than predicting speech intelligibility or perceptual benefit, its intended function is to characterize hearing-device-related changes across complementary spectral, segmental acoustic, and temporal domains. Its defining principle is that each ear serves as its own reference: the unaided intracanal recording constitutes the Individual Acoustic Reference (IAR), whereas the aided recording represents the processed condition. In the intended application, hearing device-related transformations are expressed through direct paired comparison of unaided and aided recordings obtained from the same ear using the same acquisition pathway and settings (Figure 1).

This within-ear design extends the conceptual logic of Real-Ear Insertion Gain, which quantifies the level change produced by a hearing device relative to the unaided ear, to a multidimensional description of the acoustic organization of speech [14]. The individual unaided recording incorporates the acoustic effects of the listener’s external ear and the standardized measurement pathway, thereby providing the baseline against which a subsequent aided recording is intended to be interpreted.

The validity of this approach depends on a necessary preliminary condition: the unaided recording must itself be sufficiently stable and reproducible. If the IAR varies substantially across otherwise equivalent acquisitions, an observed aided–unaided difference cannot be confidently attributed to hearing-device processing. Establishing the reliability of the unaided IAR must therefore precede evaluation of device-induced acoustic transformations.

Accordingly, the present study comprised three complementary methodological investigations. Study 1 characterized descriptor distributions and between-recording variability in a separate dataset of 26 standardized unaided ear recordings. Study 2 evaluated within-ear test–retest repeatability in a separate cohort in which unaided recordings were repeated during different sessions, including probe-tube removal and reinsertion. The two datasets were analyzed separately because they addressed different questions: descriptor variability across standardized ear recordings and within-ear repeatability across separate sessions. The third investigation evaluated the technical performance of the external recording pathway through electrical and acoustic tests. Aided–unaided transformations and their relationships with perceptual or clinical outcomes were outside the scope of the present study.

## 2. Materials and Methods

### 2.1. Study Design

This investigation evaluated the stability and test–retest repeatability of the unaided intracanal recording used as the Individual Acoustic Reference (IAR) in the SpettroREM method. It was not designed to assess the performance of a specific hearing device, aided–unaided acoustic transformations, or clinical outcomes.

**Figure 2:**
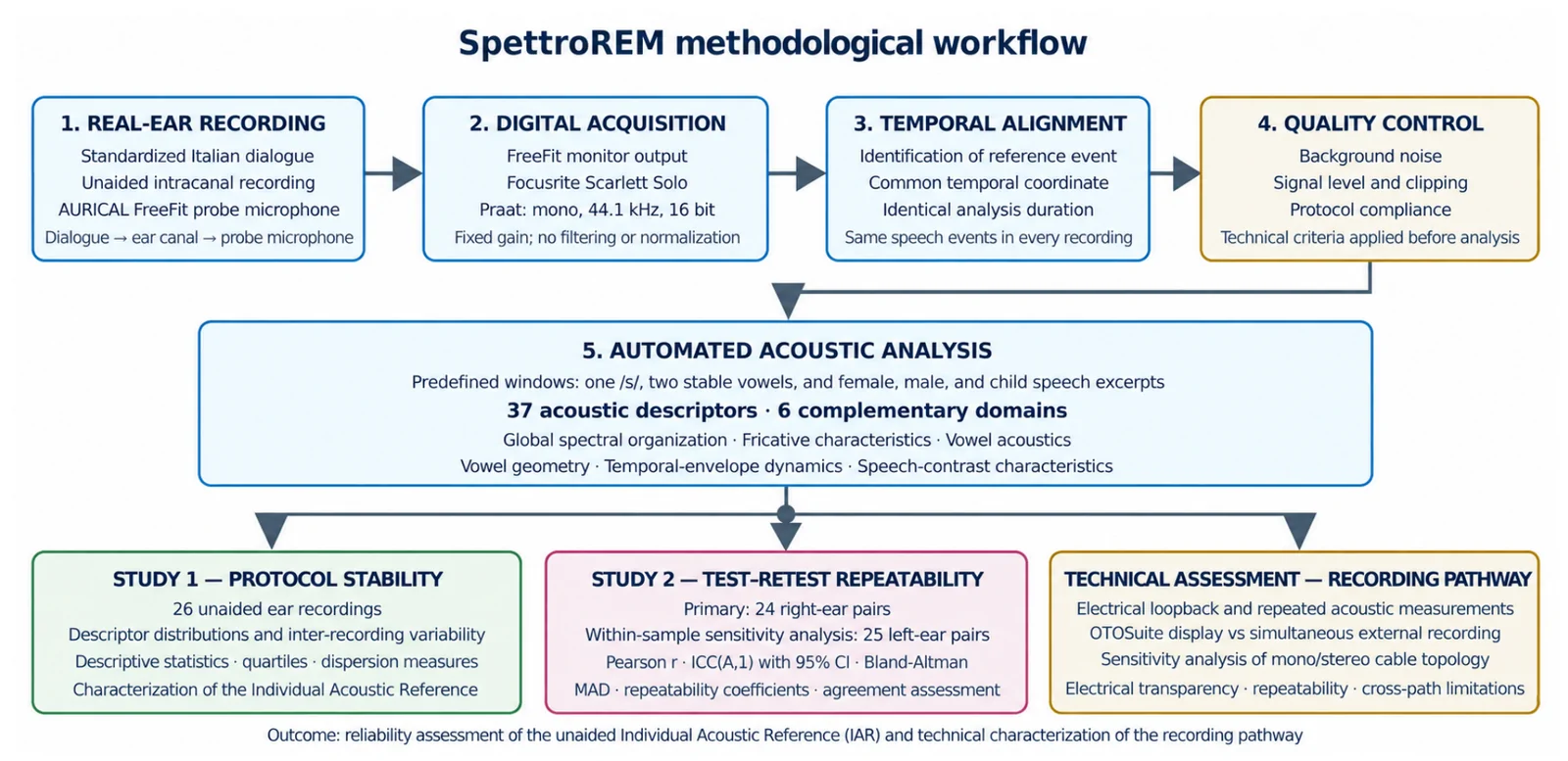
SpettroREM methodological framework. Standardized unaided intracanal recordings were acquired using the AURICAL FreeFit system and captured through the external audio interface–Praat pathway. Following temporal alignment and quality control, 37 acoustic descriptors were extracted automatically across six complementary domains. Study 1 characterized descriptor distributions and variability across 26 unaided ear recordings. Study 2 evaluated test–retest repeatability using 24 right-ear pairs as the primary dataset and 25 contralateral left-ear pairs from the same participant cohort as a separate within-sample sensitivity analysis. The technical assessment evaluated electrical transparency, acoustic repeatability, differences between the OTOsuite display and the simultaneous external recording, and the sensitivity of the results to mono and stereo cable topology. No data from the three components were pooled.

Figure 2 summarizes the complete methodological framework, comprising standardized REM acquisition, external digital recording of the probe-microphone signal, temporal alignment, quality control, automated extraction of the 37 acoustic descriptors, the two analytical studies, and the electrical and acoustic assessment of the recording pathway.

The investigation comprised two complementary analytical components and a technical assessment of the recording pathway:

Study 1—Protocol stability: characterization of the distribution and variability of the acoustic descriptors across 26 unaided ear recordings acquired according to the standardized SpettroREM protocol. This dataset was used descriptively and was not intended to estimate independent participant-level variability or population-based normative values.

Study 2—Test–retest repeatability: evaluation of paired unaided recordings obtained bilaterally from 25 participants during separate recording sessions. The primary analysis included 24 right ears because one right ear was excluded owing to tympanic membrane perforation. The 25 contralateral left ears from the same participants were analyzed separately as a within-sample sensitivity analysis to assess the robustness of the principal reliability pattern to the choice of ear used for the primary analysis. Right- and left-ear observations were not pooled.

Technical assessment of the recording pathway: evaluation of the electrical and acoustic performance of the AURICAL FreeFit–external audio interface–Praat acquisition pathway. Electrical loopback testing assessed the cable–audio interface–Praat segment, whereas repeated acoustic measurements characterized within-path repeatability and compared the OTOsuite display with the simultaneous external recording obtained from the FreeFit monitoring output. A secondary post-study bridge experiment assessed electrical transparency and bilateral acoustic behavior using an alternative stereo cable configuration. These bridge data were analyzed separately and were not combined with the study datasets.

The ear was treated as the measurement unit because the method characterizes the acoustic signal recorded within an individual external auditory canal. All 26 ear recordings were retained in Study 1 because this component provided a descriptive characterization of variability across standardized recordings and did not involve participant-level inferential analysis. For the primary test–retest analysis, only one ear per participant was included to preserve participant-level statistical independence. The contralateral left ears were analyzed separately and used only to determine whether the principal reliability pattern was reproduced on the opposite side.

The datasets were analyzed according to their distinct methodological purposes. Study 1 characterized descriptor distributions and variability across standardized unaided ear recordings, Study 2 assessed within-ear agreement between repeated recordings, and the technical assessment characterized the performance and limitations of the external acquisition pathway. No data from these components were pooled.

### 2.2. Study Datasets

#### 2.2.1. Study 1: Protocol-Stability Dataset

Study 1 comprised 26 unaided real-ear recordings, with each recorded ear treated as a separate measurement unit. Only one recording per ear was included. Where recordings from both ears of the same participant were available, they were retained as separate ear-level observations because the SpettroREM method characterizes the acoustic signal recorded within each individual external auditory canal.

This dataset was used to characterize the distribution and between-recording variability of the 37 acoustic descriptors under standardized acquisition conditions. The analyses were descriptive and did not assume that all ear-level observations represented independent participants.

The dataset was not intended to establish clinical normative values, diagnostic reference intervals, or independent estimates of intersubject variability. Its purpose was to characterize descriptor distributions across standardized unaided ear recordings and to derive preliminary technical screening limits for identifying recordings that might require quality-control review.

#### 2.2.2. Study 2: Test–Retest Dataset

Test–retest repeatability was evaluated in 25 participants who underwent bilateral unaided recordings during two separate sessions performed on different days. This produced one test–retest pair for each recorded ear.

The primary reliability analysis was based on the right ear. One right ear was excluded because of tympanic membrane perforation; the final primary dataset therefore comprised 24 right-ear test–retest pairs from 24 participants. This single-ear primary analysis avoided the participant-level statistical dependence that would have resulted from including both ears of the same participant in the same analysis.

The 25 contralateral left-ear test–retest pairs obtained from the same participant cohort were analyzed separately as a within-sample sensitivity analysis. This analysis examined whether the principal reliability pattern observed in the primary right-ear analysis was also reproduced on the opposite side. Right- and left-ear observations were not pooled, and the left ears were not treated as observations from additional independent participants.

No formal a priori sample-size calculation was performed for the test–retest study. Given its preliminary and estimation-oriented nature, the precision of the reliability estimates was explicitly quantified using 95% confidence intervals. Reliability estimates from the 24-participant primary analysis were therefore interpreted together with their confidence intervals and measures of absolute agreement rather than on the basis of ICC point estimates alone.

Between the two sessions, the probe tube was completely removed and subsequently reinserted. Probe position was reverified before the second recording using the probe-tube placement-check function of the REM system. This procedure allowed the repeatability estimates to incorporate important sources of variability encountered during routine clinical reacquisition, including probe-tube replacement and reconstruction of the measurement conditions.

The Study 2 recordings were used exclusively for the test–retest analysis and were not added to the Study 1 dataset. The two datasets were analyzed separately because they addressed different methodological objectives: Study 1 characterized descriptor distributions and variability across standardized unaided ear recordings, whereas Study 2 quantified within-ear agreement between repeated recordings.

#### 2.2.3. Technical Quality Control

Before statistical analysis, all recordings underwent technical quality-control review according to predefined acquisition-level acceptance criteria. These criteria included absence of signal clipping, adequate overall recording level, acceptable background noise, correct probe-tube positioning, complete acquisition of the standardized speech stimulus, and compliance with the standardized acquisition procedure.

Quality-control decisions were based exclusively on the technical characteristics of the recordings and were made before calculation of descriptor-level reliability estimates or assignment of descriptors to the exploratory reliability tiers. Recordings that failed the acquisition-level acceptance criteria were excluded before descriptor-level statistical analysis.

Insufficient signal energy within an individual predefined segment did not result in exclusion of the complete recording. When insufficient segment energy prevented valid calculation of a segment-dependent descriptor, that descriptor was classified as non-evaluable for the corresponding recording, and the number of evaluable test–retest pairs was reported separately.

The preliminary Tukey-fence screening limits derived from the Study 1 descriptor distributions were intended only as exploratory quality-control flags for future recordings. They were not used retrospectively to exclude recordings or descriptors from the present reliability analyses.

### 2.3. Recording System

All recordings were obtained using a conventional acoustic Real-Ear Measurement configuration based on the AURICAL FreeFit probe-microphone system and OTOsuite software (version 4.93, Natus Medical Inc.). Probe-tube calibration, loudspeaker calibration, and probe-tube placement verification were performed before each measurement session or recording series, as appropriate, in accordance with the manufacturer’s recommendations and routine clinical REM procedures.

The probe-microphone signal available at the analog monitoring output of the AURICAL FreeFit system was transferred through a mono TS 3.5-to-6.35 mm cable to a Focusrite Scarlett Solo fourth-generation USB audio interface. The signal was then recorded digitally on a computer using Praat version 6.4.25 [15].

The complete recording chain was as follows:

Speech stimulus → loudspeaker → external auditory canal → probe microphone → AURICAL FreeFit → analog monitoring output → mono TS cable → Focusrite Scarlett Solo → computer → Praat.

The external recording pathway was introduced exclusively to obtain a digital representation of the probe-microphone signal for subsequent offline acoustic analysis. Sound-pressure-level calibration remained the responsibility of the AURICAL FreeFit REM system; the external audio interface and Praat pathway did not provide an independently calibrated SPL measurement. SpettroREM should therefore be regarded as an analytical extension of conventional calibrated REM rather than as an alternative REM system.

Recordings were acquired in mono at a sampling rate of 44.1 kHz and 16-bit resolution. The audio-interface input gain and all other acquisition settings were kept fixed across study recordings. No automatic gain control, equalization, filtering, noise reduction, signal-level normalization, or other post-acquisition signal conditioning was applied to the study recordings before descriptor extraction. The mean normalization used specifically for the technical comparison of spectral shape between the OTOsuite and external pathways is described separately in Section 2.4 and was not applied to the recordings used for descriptor analysis.

All participating centers used the same acquisition settings and analytical workflow. Complete equipment and acquisition specifications are reported in Supplementary Table S6.

### 2.4. Validation of the External Recording Pathway

Because several SpettroREM descriptors characterize spectral properties, the external acquisition pathway was technically evaluated before the descriptor results were interpreted. The pathway used for the study remained unchanged across all recordings: the AURICAL FreeFit/OTOsuite analog monitoring output was connected through the same 3.5-to-6.35 mm mono TS cable to a Focusrite Scarlett Solo fourth-generation audio interface, and the signal was recorded in Praat as a mono waveform at 44.1 kHz and 16-bit resolution. Input gain was kept fixed, and no automatic gain control, equalization, filtering, signal-level normalization, or other post-recording signal conditioning was applied to the study recordings before descriptor extraction. All study recordings and derived SpettroREM measures were obtained through this same pathway.

Validation comprised complementary electrical and acoustic tests.

For the primary electrical loopback test, standardized broadband signals were routed through the mono cable–audio interface–Praat pathway, and the recorded spectrum was compared with the source spectrum in one-third-octave bands from 250 to 6000 Hz after removal of the overall level offset. The pathway was considered electrically transparent if all band-specific deviations remained within the prespecified ±2-dB acceptance range and no clipping was detected.

For the primary acoustic validation, probe-tube calibration was completed before each measurement series. The same right ear was measured during two separate sessions, with three repeated International Speech Test Signal (ISTS) presentations per session. OTOsuite spectra and simultaneous external Praat recordings were reduced to the same nine one-third-octave center frequencies: 250, 500, 750, 1000, 1500, 2000, 3000, 4000, and 6000 Hz. Because the two outputs originate from different processing points and are represented on different absolute scales, cross-path comparison focused on spectral shape after subtraction of the across-band mean from each spectrum. Cross-path correspondence was quantified using Pearson’s r, mean absolute error (MAE), root-mean-square error (RMSE), and maximum absolute band difference. Within-path repeatability was assessed using pairwise correlations and the maximum across-trial band range.

After completion of the primary study dataset, a secondary bridge experiment was performed using a stereo splitter cable to examine whether cable topology contributed to the observed cross-path spectral differences and to assess channel separation. Following successful probe-tube calibration, three bilateral acoustic repetitions were obtained. In addition, three electrical loopback recordings were obtained for each of two distinct broadband stimuli, pink noise and white noise. The secondary bridge data were analyzed separately and were not used to recalculate any primary SpettroREM descriptor or reliability estimate. They served exclusively as a post-study sensitivity analysis of cable topology, electrical transparency, and preservation of ear-related differences.

### 2.5. Speech Stimulus

The stimulus was a standardized Italian conversational dialog containing background noise, identified as Dialogue 31 in the AURICAL FreeFit/OTOsuite stimulus library. It included speech produced by an adult female speaker, an adult male speaker, and a child.

Connected conversational speech was selected because it provides a more naturalistic speech sample than isolated tones, words, or phonetic segments and contains multiple predefined events suitable for global spectral, segmental acoustic, and temporal-envelope analysis within a single presentation.

The same stimulus and presentation settings were used for every study recording. The dialog was presented through the calibrated REM loudspeaker at a nominal level of 65 dB SPL measured at 1 m. Its duration was approximately 44 s; consequently, acquisition of the unaided IAR required less than one minute of recording time, excluding routine REM preparation, calibration, and probe-tube positioning.

### 2.6. Individual Acoustic Reference

The unaided intracanal speech recording was defined as the Individual Acoustic Reference (IAR). In the intended application of SpettroREM, each aided recording is compared exclusively with the unaided recording obtained from the same ear using the same speech stimulus, REM system, recording pathway, acquisition settings, and analytical procedure.

The IAR therefore provides an individualized acoustic baseline that incorporates the anatomical filtering characteristics of the external ear together with the characteristics of the standardized measurement pathway. It is not a population-based normative reference, a prescriptive target, or an independently calibrated SPL measurement.

The present investigation evaluated only the stability and test–retest repeatability of this unaided reference. No aided–unaided comparisons were performed, and the validity of using the IAR to characterize hearing device-induced transformations remains to be established directly.

### 2.7. Temporal Alignment

Before acoustic analysis, each recording was temporally aligned in Praat using the first reproducible high-energy vowel occurring near the beginning of Dialogue 31 as the common acoustic landmark. The operator simultaneously displayed the waveform, spectrogram, and intensity contour and identified the intensity maximum of the selected vowel. The complete recording was then shifted so that this intensity maximum occurred at exactly 1.000 s.

Following alignment, each recording was truncated to retain the same 41.35 s analysis interval, corresponding to speech material extending to 43.87 s in the original stimulus. This procedure retained the same portion of the standardized dialog at common temporal coordinates across recordings. Alignment involved only a uniform temporal shift and truncation; no time stretching, temporal warping, resampling, or signal modification was applied.

Temporal alignment was operator-guided and constituted the only manual signal-positioning step. Following alignment and technical quality-control review, the predefined coordinates of the selected fricative, vowel, and speaker-specific segments were applied automatically by the Praat script without further manual adjustment. No descriptor was selected or measured manually during the alignment procedure. All recordings were acquired at the participating centers according to the standardized protocol, but temporal alignment and descriptor extraction were performed centrally by a single investigator (S.B.) for the entire dataset. Between-operator variability in landmark identification therefore did not contribute to the present estimates. Because the two sessions of each test–retest pair were aligned independently, any within-operator variability in landmark placement is already incorporated into the reported repeatability estimates rather than excluded from them.

This approach minimized differences in descriptor extraction caused by variation in recording onset and positioned the same predefined speech events within the corresponding analytical windows. However, the intra- and inter-operator repeatability of landmark identification was not formally assessed in the present study.

**Figure 3:**
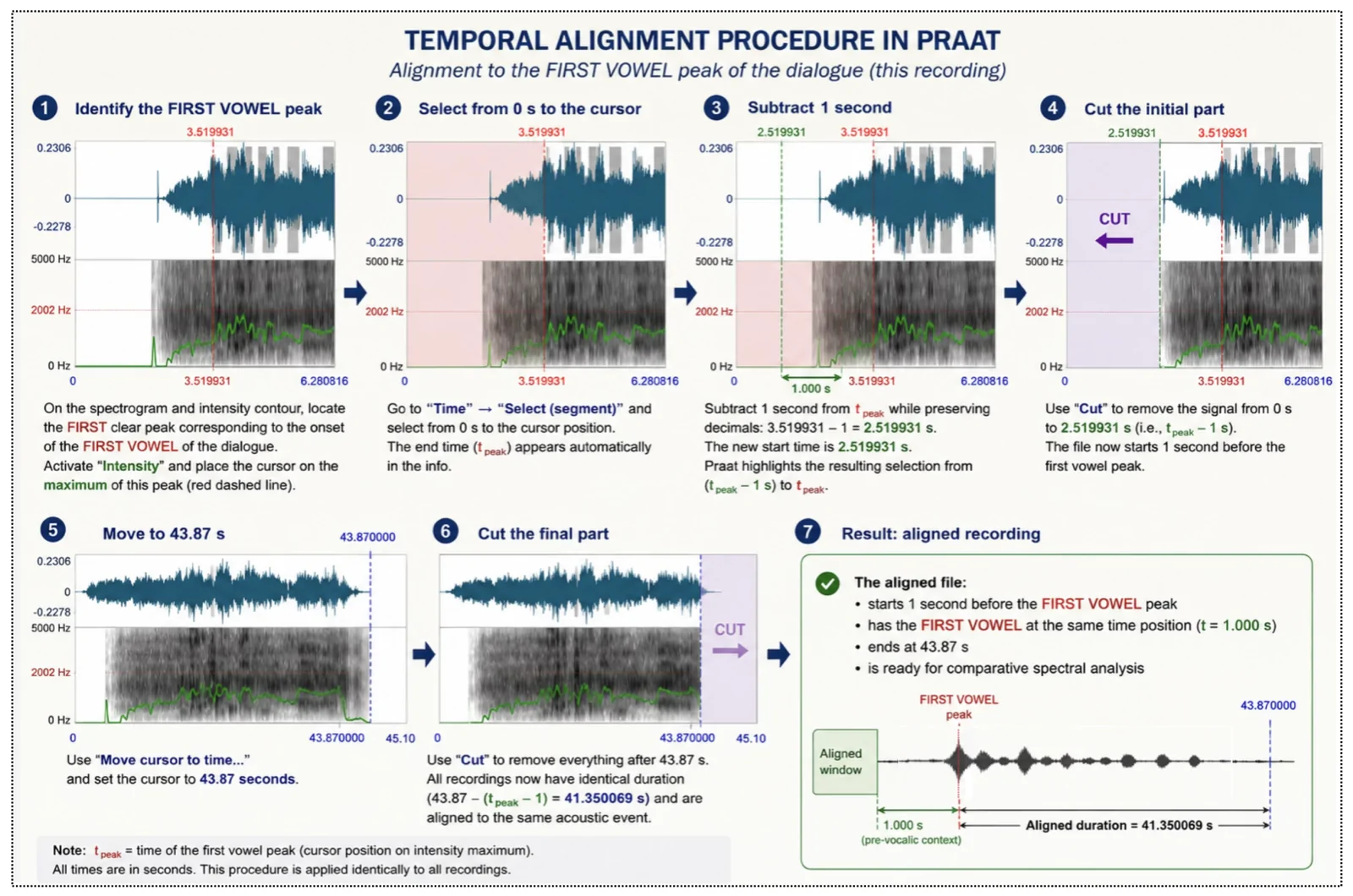
Temporal-alignment procedure applied to the SpettroREM recordings in Praat. The operator identified the intensity maximum of the first reproducible high-energy vowel near the beginning of Dialogue 31. The recording was shifted so that this landmark occurred at 1.000 s and was then truncated to retain a common 41.35 s analysis interval. Alignment involved only temporal shifting and truncation; no time stretching, temporal warping, resampling, or signal modification was applied. Following alignment, all analytical windows were positioned automatically by the Praat script.

Figure 3 illustrates the alignment landmark and its intensity maximum, the 1.000 s reference coordinate, and the resulting common analysis interval.

### 2.8. Selection, Development, and Organization of the Acoustic Descriptors

The SpettroREM descriptor set was developed through a progressive, hypothesis-driven procedure conducted between October 2025 and June 2026. The measures were not selected as a predefined battery or imported as a complete set from the literature. Instead, the library accumulated progressively as different acoustic properties of the standardized speech stimulus were identified, operationalized, and incorporated into successive versions of the automated Praat scripts. Development and selection of the 37-descriptor panel were completed without reference to the formal test–retest reliability results reported in the present study.

Development began with four unaided ear-canal recordings obtained using the standardized speech stimulus. These recordings were examined through auditory inspection and visual analysis of the waveform, spectrogram, intensity contour, temporal envelope, formant structure, and background-noise characteristics. Their qualitative similarity indicated that recurring acoustic information could be identified consistently and supported the feasibility of developing an automated extraction procedure. This preliminary comparison was exploratory and was not intended as a formal stability or test–retest assessment.

The initial quantitative core consisted of established acoustic variables available in Praat, including intensity, formant frequencies, harmonic-to-noise ratio (HNR), long-term spectral measures, and spectral centroid. These variables provided direct physical descriptions of recording level, vowel resonances, periodicity, and spectral-energy distribution. The framework was subsequently extended to acoustic dimensions considered potentially susceptible to hearing-device processing but not sufficiently represented by the initial measures. These extensions included frequency-band energy distribution, high-frequency fricative characteristics, local segmental measurements, relationships between vowel segments, temporal-envelope dynamics, and intensity organization within the continuous speech stimulus.

The measures were organized into six complementary analytical domains: Global LTASS Organization, Fricative Spectral Characteristics, Vowel Acoustics, Vowel Geometry, Temporal-Envelope Dynamics, and Speech-Level and Contrast Characteristics (Table 1). The choice of these domains was informed by established evidence concerning long-term speech spectra [16], vowel and formant acoustics [17,18], fricative acoustics [19,20], and temporal-envelope processing [21,22]. The literature therefore supported the acoustic dimensions to be represented, whereas the specific combination, adaptation, and computational implementation of the measures were developed within the SpettroREM framework.

Global LTASS Organization was initially represented by eight measures derived from the long-term spectrum of the complete recorded speech signal: LTASS peak level, LTASS peak frequency, global spectral centroid, spectral spread, mean energy in three predefined frequency bands (0–1, 1–4, and 4–8 kHz), and the derived LTASS high-to-low-frequency ratio. Together, these measures characterized the overall distribution of speech energy and the balance between low- and high-frequency spectral components.

Three reproducibly identifiable speech events were selected from the standardized stimulus: an /s/ fricative and two vowel segments, V1 and V2. Local measurements of these events were calculated across five overlapping 80 ms analysis windows centered at −40, −20, 0, +20, and +40 ms relative to each predefined marker. Valid estimates from the five windows were averaged to reduce dependence on a single analysis frame, local spectral fluctuations, and small residual alignment differences. The five windows contributed to one final estimate for each descriptor and recording and were not treated as independent observations.

Six measures were initially calculated for the selected /s/: intensity, high-to-low-frequency energy ratio, HNR, robust spectral centroid, spectral slope, and spectral brightness. Fourteen vowel measures were initially obtained by calculating F1, F2, F3, intensity, HNR, aperiodicity, and spectral centroid separately for V1 and V2. Because hearing-device processing may also affect acoustic relationships between speech segments, six Vowel Geometry measures were derived from the window-averaged V1 and V2 values: ΔF1, ΔF2, vowel distance F1–F2, Δcentroid, extended vowel distance, and standardized inter-vowel distance. These relational measures characterized the separation and relative organization of the two vowel segments rather than the properties of either vowel considered independently.

Four temporal-envelope measures were added because time-averaged spectral measures do not describe the evolution of signal amplitude: the 10–90% attack time of /s/, the 10–90% attack time of V1, and the post-vowel decay times to −3 and −6 dB. These measures were calculated from the continuous intensity contour within predefined temporal intervals and did not use the five-window segmental procedure.

The Speech-Level and Contrast Characteristics domain comprised six measures describing two complementary aspects of intensity organization within the standardized stimulus. Female-, male-, and child-speaker P90–P10 values quantified the robust within-speaker intensity range over three predefined speech intervals. These intervals were analyzed separately because they differed in baseline intensity distribution, spectral composition, and prosodic characteristics and could therefore respond differently to level-dependent, frequency-dependent, or adaptive hearing-device processing. Pooling the three speakers into a single measure could conceal speaker-specific changes in the internal intensity variation of the stimulus.

The remaining three measures in this domain quantified the local recording level of predefined /s/, Vowel1, and Vowel2 events. They were calculated as energy-mean intensities over dedicated 80 ms windows centered on speech events distinct from those used for the five-window fricative and vowel analyses. These measures provided local consonantal and vocalic level references within the continuous speech stimulus and were not themselves measures of speech contrast. The broader designation “Speech-Level and Contrast Characteristics” was therefore used to distinguish the three within-speaker P90–P10 contrast measures from the three local-level measures.

This progressive procedure produced an exploratory library of 44 measures: eight Global LTASS Organization measures, six Fricative Spectral Characteristics measures, 14 Vowel Acoustics measures, six Vowel Geometry measures, four temporal-envelope measures, and six Speech-Level and Contrast Characteristics measures. Thus, 44 was neither an a priori target nor a statistically determined number; it was the total number of measures implemented by the end of the exploratory development phase. Candidate measures were included when they represented a physical acoustic property that hearing-device processing could plausibly modify and could be extracted objectively and automatically from reproducibly identifiable portions of the standardized stimulus using deterministic Praat procedures.

During final construction of the IAR framework, the 44 exploratory measures were reviewed according to their roles in representing the targeted acoustic dimensions. Thirty-seven were designated as the primary descriptor library, whereas seven were retained as accessory exploratory outputs because they were derived from, or partially overlapped with, information already represented by the primary descriptors. The seven accessory measures were the LTASS high-to-low-frequency ratio, robust spectral centroid and spectral slope of /s/, HNR of V1, HNR of V2, Δcentroid, and extended vowel distance. The LTASS high-to-low-frequency ratio was derivable from the primary LTASS band measures; robust spectral centroid and spectral slope provided alternative representations of fricative spectral distribution; the two vowel HNR measures partially overlapped with the representation of vowel periodicity provided by the corresponding aperiodicity measures; and Δcentroid and extended vowel distance were relational measures that partially overlapped with the primary vowel and Vowel Geometry descriptors.

The final primary panel therefore comprises 37 descriptors organized into the same six domains: Global LTASS Organization, seven descriptors; Fricative Spectral Characteristics, four; Vowel Acoustics, 12; Vowel Geometry, four; Temporal-Envelope Dynamics, four; and Speech-Level and Contrast Characteristics, six. The developmental origin, extraction procedure, acoustic rationale, measurement unit, methodological relevance, and final classification of all 44 exploratory measures are reported in Supplementary Table S1.

The transition from 44 exploratory measures to 37 primary descriptors did not result from automated statistical feature selection or retrospective exclusion of measures showing unfavorable reliability. It was based on conceptual role, derivability, and partial informational overlap and was completed without using the formal reliability estimates obtained from the Study 2 test–retest dataset. The final panel should therefore be considered a transparent first implementation of the SpettroREM conceptual framework rather than a statistically optimized, minimal, or fully non-redundant feature set. Formal procedures such as principal-component analysis, clustering, or multivariable feature reduction were not used to define the current descriptor panel.

The subsequent classification of the 37 primary descriptors into Core, Candidate, and Secondary reliability tiers was a separate exploratory post hoc analysis. Because the same Study 2 dataset was used both to estimate descriptor reliability and to assign descriptors to these tiers, their stability and generalizability require confirmation in an independent sample. The tier classification should therefore be regarded as hypothesis-generating and not as a further reduction or definitive validation of the 37-descriptor library.

### 2.9. Automated Acoustic Analysis and Workflow

**Figure 4:**
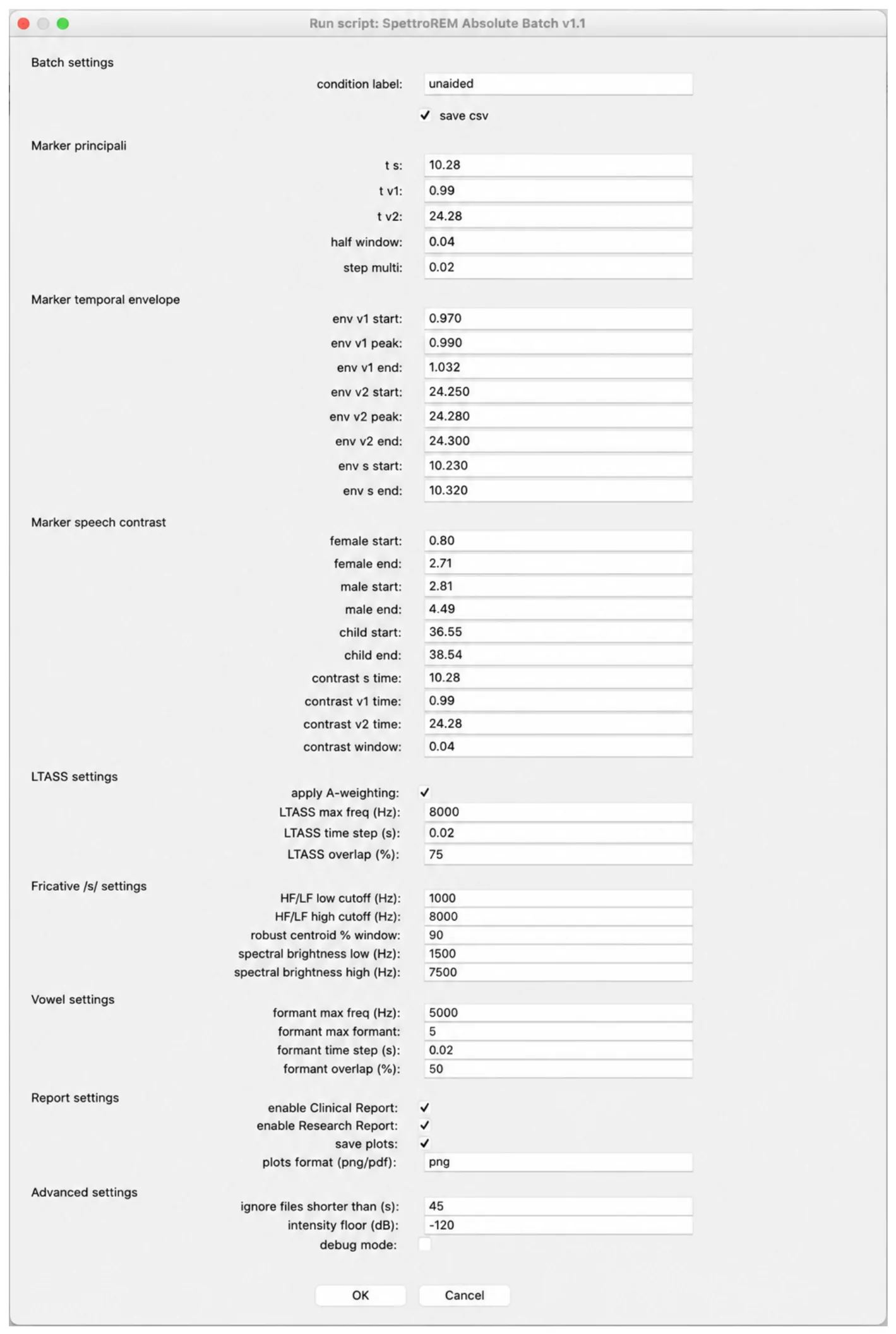
Screenshot of the analysis script window showing the default values used for the evaluations on the recorded dialog.

Each recording was processed in Praat using a dedicated SpettroREM script (Figure 4). The complete workflow comprised the following sequential stages:

- Import of the mono WAV recording;
- Verification of recording duration and technical compatibility;
- Operator-guided temporal alignment to the predefined acoustic landmark;
- Truncation to the common analysis interval;
- Application of the fixed global, segmental, and temporal analysis windows;
- Automated extraction of the 37 primary acoustic descriptors;
- Classification of individual segment-dependent descriptors as non-evaluable when insufficient signal energy prevented valid estimation;
- Organization of the descriptor outputs into the six analytical domains;
- Export of descriptor-level results to a structured CSV file;
- Separate statistical analysis of Study 1 descriptor distributions or Study 2 test–retest repeatability, according to the dataset.

After the operator completed temporal alignment and technical quality-control review, application of the analytical windows, descriptor extraction, organization of the outputs, and data export were automated and required no manual acoustic measurement. Statistical analyses were performed separately from the Praat extraction workflow, as described in Section 2.10.

The complete recording, alignment, acoustic-analysis, and statistical workflow is summarized in Figure 2.

### 2.10. Statistical Analysis

Study 1 and Study 2 were analyzed separately because they addressed different methodological objectives.

No formal a priori sample-size calculation was performed for the test–retest study. Given its preliminary and estimation-oriented nature, the precision of the reliability estimates was explicitly quantified using 95% confidence intervals. The primary analysis included 24 participants, each contributing one evaluable right-ear test–retest pair. Reliability estimates were therefore interpreted together with their confidence intervals and measures of absolute agreement rather than on the basis of ICC point estimates alone.

For Study 1, the distribution of each descriptor was summarized using the mean, standard deviation, median, first and third quartiles, minimum, and maximum. The coefficient of variation was calculated only where mathematically appropriate as a descriptive measure of relative dispersion. Because coefficients of variation can be unstable or difficult to interpret for signed variables, variables with means close to zero, and logarithmic measures, they were not calculated for these descriptors and were not used as the sole criterion for protocol stability. Preliminary technical screening limits were calculated using Tukey fences, defined as Q1 − 1.5 × IQR and Q3 + 1.5 × IQR, where IQR = Q3 − Q1. Study 1 analyses were descriptive and did not assume that all ear-level observations represented independent participants.

For Study 2, the primary test–retest analysis was performed on 24 right ears, with one ear contributed by each participant. The 25 contralateral left ears from the same participant cohort were analyzed separately as a within-sample sensitivity analysis. Right- and left-ear observations were not pooled, and the left ears were not treated as observations from additional independent participants. The contralateral analysis was used to examine whether the principal reliability pattern observed in the primary right-ear analysis was reproduced on the opposite side.

Test–retest association was assessed for each descriptor using Pearson’s correlation coefficient. Its 95% confidence interval was calculated using Fisher’s z transformation. Test–retest reliability was assessed using the single-measure, absolute-agreement intraclass correlation coefficient based on a two-way random-effects model [ICC(A,1), equivalent to ICC(2,1)]. The 95% confidence intervals for ICC(A,1) were estimated using 10,000 bootstrap resamples at the participant level.

Absolute agreement was further characterized using the mean test–retest difference, mean absolute difference (MAD), Bland–Altman bias and 95% limits of agreement [23], and the repeatability coefficient (RC). Differences were calculated as Session 2 minus Session 1. The 95% confidence interval of the bias was calculated using the *t* distribution. The limits of agreement were calculated as the mean difference ± 1.96 × the standard deviation of the differences, and RC was calculated as 1.96 × the standard deviation of the differences, corresponding to half the width of the limits of agreement.

ICC point estimates were described using the conventional categories proposed by Koo and Li [24]: poor, <0.50; moderate, 0.50–0.74; good, 0.75–0.89; and excellent, ≥0.90. These labels were used descriptively and were interpreted together with the 95% confidence intervals and measures of absolute agreement rather than as definitive thresholds.

To facilitate exploratory descriptor-level interpretation, the 37 descriptors were also assigned post hoc to Core, Candidate, and Secondary tiers using a hierarchical procedure. Core comprised Global LTASS Organization descriptors whose lower 95% ICC confidence limit was ≥0.75. Among the remaining descriptors, Candidate comprised non-level-dependent descriptors with an ICC point estimate ≥0.75 but a lower confidence limit <0.75. Secondary comprised all remaining descriptors, including directly recording-level-dependent local intensity measures regardless of their ICC point estimates and descriptors with ICC <0.75. This classification was exploratory, post hoc, and hypothesis-generating. Because the same Study 2 dataset was used both to estimate descriptor reliability and to assign descriptors to tiers, the stability and generalizability of this classification require confirmation in an independent sample.

All analyses were performed at the individual-descriptor level. When a descriptor could not be calculated because the relevant segment contained insufficient signal energy, the observation was classified as non-evaluable rather than assigned an artificial value. Reliability and agreement estimates were calculated using the available complete test–retest pairs for that descriptor, and the corresponding number of evaluable pairs was reported.

This study did not apply multivariate feature selection, principal-component analysis, clustering, or predictive modeling. The tier classification did not constitute a formal feature-selection procedure.

## 3. Results

### 3.1. Measurement-System Validation

The electrical loopback test of the primary mono pathway used for this study met the prespecified ±2-dB acceptance criterion over 250–6000 Hz, with no evidence of clipping. This indicated that the mono cable–audio-interface–Praat segment did not introduce a material frequency-dependent alteration under the tested settings. The post-study stereo electrical bridge corroborated this finding: across three pink-noise and three white-noise recordings, the maximum individual one-third-octave deviation from the corresponding source signal was 0.091 dB.

The complete acoustic pathway showed high within-path repeatability. In the primary mono-cable validation, within-path pairwise correlations ranged from 0.958 to 0.985 for the OTOsuite spectra and from 0.976 to 0.996 for the external Praat spectra. The maximum across-trial band range was 2.11–3.85 dB across sessions and pathways.

After removal of the across-band mean, cross-path spectral-shape correlations ranged from 0.847 to 0.913. However, the mean absolute difference between the mean-normalized spectral shapes ranged from 3.06 to 4.48 dB, and the maximum absolute band difference reached 11.74 dB. Thus, the OTOsuite and external pathways showed broadly corresponding spectral organization and high repeatability within each pathway, but their spectral outputs were not numerically interchangeable.

The post-study stereo bridge did not eliminate the cross-path spectral differences. Within-path repeatability of the external recordings remained very high for both ears (r = 0.996–0.998). Cross-path correlations ranged from 0.790 to 0.850 for the right ear and from 0.882 to 0.901 for the left ear, with maximum absolute band differences of 9.43–12.91 dB. In the tested bilateral measurement, the interaural spectral-difference pattern derived from the external recordings corresponded to that obtained from OTOsuite (r = 0.850; MAE = 0.704 dB).

Together, these findings indicate that the recording pathway was electrically transparent and acoustically repeatable under the tested settings. They also show that the external pathway preserved the observed interaural spectral-difference pattern in the secondary bilateral experiment. However, the OTOsuite display and the monitor-output-derived waveform represent distinct processing outputs and should not be treated as numerically interchangeable measurements. Complete technical-validation results are reported in the Supplementary Technical Validation Section.

### 3.2. Protocol Stability of the Individual Acoustic Reference

The first objective was to characterize the distribution and between-recording variability of the acoustic descriptors obtained using the standardized unaided recording protocol. Complete descriptive statistics for all 37 descriptors, including means, standard deviations, medians, quartiles, observed ranges, and provisional quality-control limits based on Tukey fences, are reported in Supplementary Tables S2 and S3.

**Figure 5:**
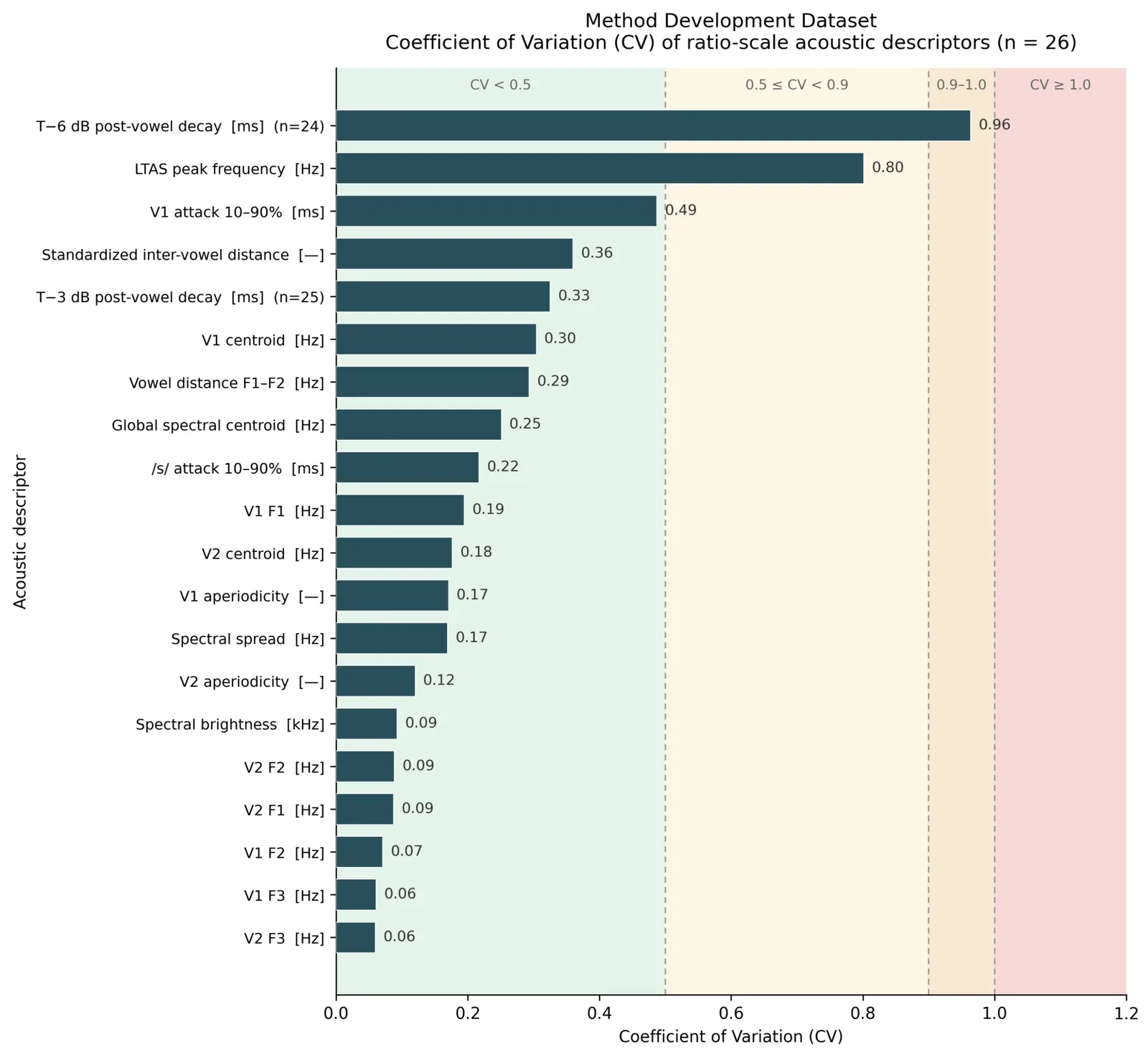
Coefficients of variation of ratio-scale acoustic descriptors in the Method-Development Dataset. Descriptors are shown in descending order of CV. The analysis included 26 unaided ear recordings, except for T−3 dB post-vowel decay (n=25) and T−6 dB post-vowel decay (n=24), owing to insufficient signal energy in the corresponding segments. CVs were calculated only for descriptors measured on positive ratio scales. Descriptors expressed on logarithmic (dB) scales or as signed differences were characterized using the descriptive statistics reported in Supplementary Tables S2 and S3. Shaded regions and vertical dashed lines at 0.50, 0.90, and 1.00 are provided solely as descriptive visual guides and should not be interpreted as validated decision thresholds.

Coefficients of variation are shown in Figure 5 only for descriptors measured on positive ratio scales, including frequency and temporal measures and positive dimensionless ratios for which the CV provides an interpretable measure of relative dispersion. Descriptors for which the CV was inappropriate because of signed values, means close to zero, or logarithmic measurement scales were characterized using the distributional statistics reported in the Supplementary Tables. In Figure 5, the descriptors are presented in descending order of CV.

### 3.3. Test–Retest Reliability of the Individual Acoustic Reference

The second objective was to evaluate the test–retest repeatability of the Individual Acoustic Reference under standardized clinical reacquisition conditions. The Repeatability Dataset comprised bilateral unaided recordings obtained from 25 participants during two sessions performed on different days and was analyzed separately from the Method-Development Dataset.

The primary analysis included 24 right-ear test–retest pairs from 24 participants. One right ear was excluded because of tympanic membrane perforation. The 25 contralateral left-ear pairs from the same participant cohort were analyzed separately as a within-sample sensitivity analysis to examine whether the principal reliability pattern observed in the primary right-ear analysis was reproduced on the opposite side. Right- and left-ear observations were not pooled.

**Figure 6:**
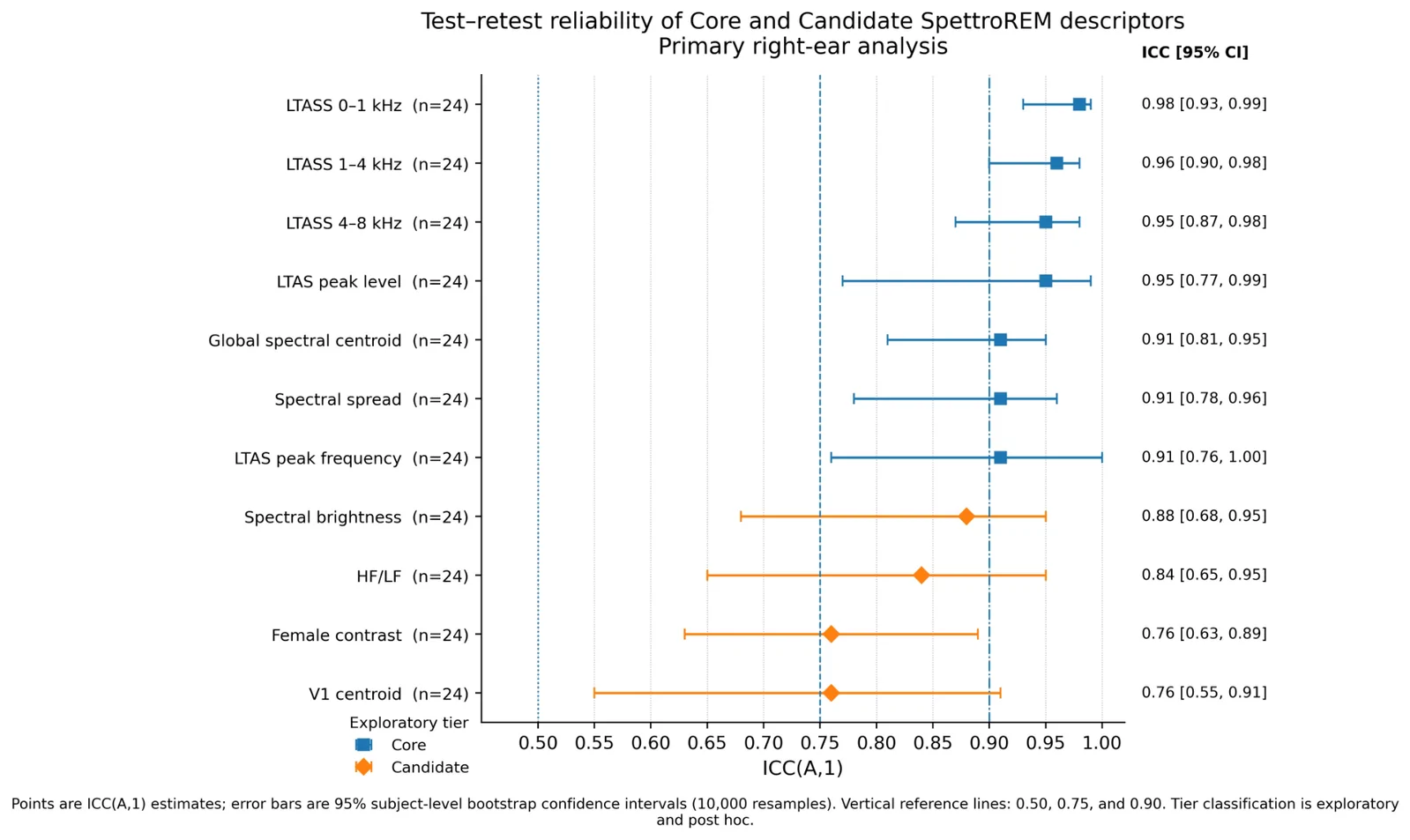
Test–retest reliability of descriptors assigned to the exploratory Core and Candidate tiers in the primary right-ear analysis. Points represent ICC(A,1) estimates, and error bars represent 95% participant-level bootstrap confidence intervals based on 10,000 resamples. Vertical reference lines indicate ICC values of 0.50, 0.75, and 0.90. Core comprised Global LTASS Organization descriptors whose lower 95% ICC confidence limit was at least 0.75. Among the remaining descriptors, Candidate comprised non-level-dependent descriptors with an ICC point estimate of at least 0.75 but a lower confidence limit below 0.75. Tier assignment was exploratory and post hoc. Complete results for all 37 descriptors are reported in Supplementary Table S4 and Supplementary Figure S1.

ICC(A,1) point estimates and their 95% participant-level bootstrap confidence intervals for the descriptors assigned to the exploratory Core and Candidate tiers are shown in Figure 6. Complete descriptor-level Pearson correlation coefficients, ICC(A,1) estimates, 95% confidence intervals, and numbers of evaluable pairs are reported in Supplementary Table S4. ICC(A,1) estimates and their 95% confidence intervals for all 37 descriptors are displayed in Supplementary Figure S1. Absolute-agreement statistics are reported in Supplementary Table S5.

In the primary right-ear analysis, the seven descriptors assigned post hoc to the Core tier showed excellent ICC(A,1) point estimates, ranging from 0.906 to 0.984, with the lower bound of the 95% confidence interval remaining ≥0.75 for every Core descriptor. Mean absolute test–retest differences for the LTASS band levels were 0.31 dB at 0–1 kHz, 0.50 dB at 1–4 kHz, and 0.88 dB at 4–8 kHz. These findings indicate that the broad spectral distribution of the unaided intracanal signal was reproduced consistently across the two recording sessions.

Four additional non-level-dependent descriptors had ICC(A,1) point estimates ≥0.75 but lower 95% confidence limits below 0.75 and were assigned to the exploratory Candidate tier. The remaining 26 descriptors were classified as Secondary because their ICC point estimates were below 0.75 or because they represented directly recording-level-dependent local intensity measures. Consequently, tier assignment should not be interpreted as a simple ranking based exclusively on ICC point estimates.

The contralateral sensitivity analysis reproduced the high repeatability of the three LTASS band levels, with ICCs ranging from 0.835 to 0.940. Spectral spread and LTASS peak level also showed good-to-excellent agreement, whereas global spectral centroid showed moderate-to-good agreement and LTASS peak frequency was not reproducible. Thus, the broad long-term spectral pattern was also observed on the opposite side within the same participant cohort, but the contralateral analysis did not constitute an independent replication and did not support uniform repeatability across every descriptor in the global spectral domain.

Repeatability was not uniform across the complete descriptor set. Several segmental, vowel-derived, recording-level-dependent, and temporal-envelope descriptors showed lower or more uncertain agreement. Descriptors requiring sufficient energy within short predefined segments could not always be calculated. In these cases, the individual measurement was classified as non-evaluable rather than assigned an artificial value or automatically considered unreliable. Reliability estimates were therefore based on the available complete test–retest pairs for each descriptor.

ICC point estimates were interpreted together with their 95% confidence intervals and measures of absolute agreement. Bland–Altman bias and limits of agreement, mean absolute differences, and repeatability coefficients are reported in Supplementary Table S5. The exploratory Core, Candidate, and Secondary classification was derived from the same dataset used to estimate reliability and must therefore be confirmed in an independent sample. The findings support repeatability principally for global spectral organization, particularly the LTASS band levels, and for selected additional descriptors under the standardized acquisition conditions used in this study; they do not establish repeatability of the complete multidomain descriptor library.

### 3.4. Behavior of Level-Dependent Descriptors

The reliability analysis showed that repeatability differed according to the physical property represented by each descriptor. Measures describing the broad spectral distribution of speech were generally the most repeatable, whereas several recording-level-dependent intensity measures showed greater between-session variability. Temporal-envelope and segmental descriptors exhibited more heterogeneous results and cannot be considered uniformly repeatable.

This pattern should be interpreted in relation to the acquisition architecture described in Section 2.3 and the technical validation reported in Section 2.4. Absolute sound-pressure-level calibration was provided by the REM system, whereas the external digital recording pathway did not provide an independently calibrated SPL measurement. Consequently, descriptors that depend directly on recording level may be affected by small differences in acquisition gain, probe-tube positioning, or reconstruction of the measurement conditions. By contrast, measures based primarily on spectral shape or relative energy distribution are less sensitive to uniform changes in recording level, although they may still be influenced by frequency-dependent characteristics of the acquisition pathway.

Level-dependent descriptors were retained and are reported individually rather than excluded solely on the basis of their present reliability estimates. Their values should be interpreted cautiously and primarily within paired recordings acquired using the same REM system, monitoring output, cable configuration, audio interface, gain settings, and acquisition protocol. Some local intensity descriptors showed high ICC point estimates but remained in the Secondary tier because tier assignment also considered their direct dependence on recording level and was not based exclusively on the ICC point estimate. The present findings identify global spectral measures as the most consistently repeatable domain and indicate which additional descriptors require further evaluation, but they do not establish a formally validated reliable descriptor set.

### 3.5. Descriptor-Level Interpretation

The expanded test–retest analysis showed that reliability differed across acoustic domains and individual descriptors. The most consistent results were observed for measures of global spectral organization, whereas segmental, vowel-derived, recording-level-dependent, and temporal-envelope descriptors showed more heterogeneous test–retest behavior. The contralateral sensitivity analysis reproduced the principal pattern observed for the global spectral descriptors but did not demonstrate uniformly high repeatability across the complete descriptor library. The functional organization and overall test–retest behavior of the six analytical domains are summarized in Table 1.

All 37 descriptors were retained and reported individually, including those with poor point estimates or wide confidence intervals. To aid descriptive interpretation, the descriptors were assigned post hoc to exploratory Core, Candidate, and Secondary tiers. Seven descriptors met the Core criterion, four were classified as Candidate, and 26 as Secondary. These categories summarize the present reliability results but do not constitute a validated feature-selection procedure or an intrinsic ranking of descriptor performance. Interpretation therefore considered the ICC estimates and their 95% confidence intervals together with mean absolute differences, Bland–Altman limits of agreement, and repeatability coefficients.

The Core, Candidate, and Secondary classification should be regarded as exploratory, post hoc, and hypothesis-generating. Because the same primary right-ear dataset was used both to estimate descriptor reliability and to assign descriptors to tiers, the stability and generalizability of this classification must be confirmed in an independent sample. The contralateral sensitivity analysis does not provide such independent confirmation because the left ears were obtained from the same participant cohort.

For segment-dependent descriptors, insufficient signal energy occasionally prevented valid estimation. These observations were classified as non-evaluable rather than treated as measurement errors or automatically interpreted as evidence of poor reliability. The number of evaluable test–retest pairs is therefore reported separately for each descriptor in Supplementary Tables S4 and S5. In particular, T−3 dB and T−6 dB post-vowel decay should be interpreted only when the corresponding temporal segment contains sufficient energy for valid estimation.

Some descriptors are mathematically or conceptually related and cannot be considered independent sources of evidence. Derived measures, including the inter-vowel distances, partly reflect the behavior of their component variables. Similarly, favorable repeatability does not establish that a descriptor is necessary, non-redundant, sensitive to hearing-device processing, or clinically informative.

The present analysis therefore provides a descriptor-level characterization of measurement repeatability rather than a formal feature-selection procedure. Identification of a reduced and clinically informative descriptor subset will require independent confirmation, direct aided–unaided testing, and formal analysis of redundancy, dimensionality, and relationships with perceptual or clinical outcomes. Complete descriptor-level reliability and agreement results are reported in Supplementary Tables S4 and S5.

## 4. Discussion

### 4.1. Principal Findings

This study provides preliminary evidence that selected properties of the unaided Individual Acoustic Reference can be measured repeatably under standardized acquisition conditions. The 26-ear Method-Development Dataset characterized descriptor variability across standardized unaided ear recordings, whereas the expanded test–retest dataset showed that measurement repeatability was not uniform across acoustic domains.

Global spectral descriptors, including the LTASS bands, spectral centroid, and spectral spread, showed the most consistent test–retest performance. In the exploratory post hoc classification, all seven Core descriptors had ICC(A,1) point estimates between 0.906 and 0.984, with lower 95% confidence limits ≥0.75. Four additional descriptors met the Candidate criterion because their ICC point estimates were ≥0.75, but their lower confidence limits remained below 0.75.

The contralateral sensitivity analysis reproduced the principal pattern observed for the global spectral descriptors, supporting the robustness of the main finding to the choice of ear used for the primary analysis. One descriptor departed from this pattern. LTASS peak frequency met the Core criterion in the primary right-ear analysis (ICC 0.91, 95% CI 0.76–1.00) but was not reproducible in the contralateral analysis. Unlike the band levels, this descriptor identifies the location of the spectral maximum, which takes a small number of discrete values concentrated in the low-frequency region and can shift between adjacent spectral peaks of nearly equal amplitude; its between-recording variability in the Method-Development Dataset was correspondingly high (CV 0.80). Its assignment to the Core tier follows from the prespecified hierarchical rule applied to the primary dataset and is retained here for consistency, but the discrepancy between the two sides indicates that its repeatability is less securely established than that of the LTASS band levels, and it should be interpreted with the same caution as the Candidate descriptors until confirmed in an independent sample. However, because the right- and left-ear measurements were obtained from the same participants, the contralateral analysis represents a within-sample sensitivity analysis rather than an independent replication. The relative stability of these measures is consistent with previous evidence concerning the stability of acoustic characteristics derived from extended speech material [25], although language- and stimulus-specific differences, particularly at higher frequencies, remain possible [16,26].

Vowel-derived, fricative, recording-level-dependent, and temporal-envelope descriptors showed more heterogeneous repeatability. Because the same prerecorded speech stimulus was used in both sessions, this variability cannot be attributed to differences in speech production. More plausible contributors include differences in local signal energy, small residual differences in temporal alignment and window placement, automatic formant-tracking errors, probe-tube repositioning, and reconstruction of the acquisition level and measurement conditions. Segment-dependent measures that could not be calculated because of insufficient signal energy were classified as non-evaluable rather than automatically considered unreliable.

The findings therefore do not establish the repeatability of the complete 37-descriptor library or of the IAR as a single multidimensional pattern. They support repeatability principally for global spectral organization and selected additional descriptors. The Core, Candidate, and Secondary tiers summarize the results obtained in the present primary right-ear dataset but do not constitute a validated feature-selection procedure. Because the same dataset was used both to estimate descriptor reliability and to assign descriptors to tiers, the stability and generalizability of this classification must be confirmed in an independent sample. Each descriptor also requires further evaluation before its sensitivity to hearing-device processing or clinical usefulness can be established.

### 4.2. Relation to Existing Objective Measures

SpettroREM addresses a different purpose from established objective measures of hearing-aid signal processing. Metrics such as STOI and HASPI/HASQI estimate speech intelligibility or quality by comparing a processed signal with an unprocessed reference signal [7,9,10,11]. Coherence-based measures assess signal fidelity, whereas phase-inversion techniques separate speech and noise components under controlled presentation conditions [12,13,27]. More recent non-intrusive models predict intelligibility or quality without requiring a paired clean reference, but their outputs still require validation against perceptual scores or established objective measures [28].

The intended SpettroREM application instead uses an individual unaided intracanal recording as the measured baseline for a subsequent aided recording obtained from the same ear using the same acquisition pathway and settings. Unlike clean-reference comparison methods, the IAR incorporates the anatomical filtering characteristics of the individual external ear and the standardized measurement pathway. SpettroREM does not predict speech intelligibility or perceived sound quality. Its intended function is to provide a multidimensional, descriptor-based characterization of within-ear changes in spectral distribution, segmental acoustic properties, temporal-envelope dynamics, and speech-level and contrast characteristics at the probe-microphone recording point.

SpettroREM should therefore be considered complementary to intelligibility- and quality-prediction metrics rather than an alternative to them. However, the present study evaluates only the stability and test–retest repeatability of the unaided reference. It does not establish that the descriptors are sensitive to hearing-device processing or associated with speech intelligibility, listening effort, perceived quality, or patient-reported benefit. These relationships require direct aided–unaided investigation and comparison with established objective measures and perceptual outcomes.

### 4.3. Methodological Implications

The Method-Development Dataset should not be interpreted as a source of clinical reference intervals or normative values. Its purpose was to characterize the distribution and variability of descriptors obtained using the standardized acquisition protocol. The resulting empirical distributions, summarized using quartiles and Tukey fences, were also used to generate preliminary statistical screening flags for quality control. These flags are intended only to identify recordings that may require technical review and do not define normal or abnormal auditory or clinical performance. They were not used retrospectively to exclude recordings or descriptors from the present analyses.

The intended interpretation of SpettroREM remains based on a paired within-ear comparison between an individual’s aided and unaided recordings. The repeatability demonstrated for selected properties of the unaided reference provides a necessary, but not sufficient, methodological basis for this approach. It does not by itself establish the validity of the subsequent aided–unaided comparison.

The standardized inter-vowel distance does not introduce a population-based normative reference. Its fixed constants of 100, 200, and 800 Hz for the F1, F2, and spectral-centroid differences, respectively, are predefined scaling denominators rather than sample-derived standard deviations. In this context, “standardized” denotes fixed-scale normalization of the acoustic differences between V1 and V2 within an individual recording, not statistical standardization against a reference population.

### 4.4. Practical Considerations

SpettroREM was designed to operate alongside routine clinical REM without modifying probe-tube calibration, probe-tube placement, or stimulus presentation. The current implementation adds parallel capture of the AURICAL FreeFit analog monitoring output through a mono TS cable and a standard USB audio interface. Acquisition of the unaided IAR requires approximately 44 s and therefore adds less than one minute of recording time. The only additional hardware is the external audio interface, which cost approximately less than USD 200 at the time of this study. Following operator confirmation of temporal alignment and technical quality control, descriptor extraction and data export are automated using freely available Praat software (version 6.4.25) and a dedicated script.

The present implementation was developed and technically assessed using the AURICAL FreeFit/OTOsuite–Focusrite Scarlett–Praat pathway. Equivalent performance should not be assumed for other REM systems, monitoring outputs, cable configurations, or audio interfaces without platform-specific verification. The conceptual framework could potentially be applied to different REM platforms and hearing-device technologies, but this possibility remains to be demonstrated through appropriate technical validation and direct within-ear aided–unaided studies.

The Method-Development Dataset also provides preliminary descriptor distributions and Tukey-fence limits for technical screening of future recordings. Recordings containing multiple descriptor values outside these provisional limits may be flagged for inspection or repetition. Such flags do not automatically define a recording as technically unacceptable. The screening criteria are exploratory and have not been validated in an independent dataset. Furthermore, because several descriptors are correlated, the probability of observing multiple deviations cannot be estimated using a binomial model that assumes statistical independence.

SpettroREM is intended to complement conventional REM by describing selected spectral, segmental acoustic, and temporal properties of the intracanal speech signal. However, the present study evaluates only the unaided reference. Whether the method can detect meaningful hearing-device transformations or show associations with intelligibility, listening effort, perceived quality, or patient-reported benefit requires subsequent within-ear aided–unaided validation.

### 4.5. Limitations and Future Directions

The principal limitation is that the present study evaluates only the stability and test–retest repeatability of the unaided Individual Acoustic Reference. It does not assess aided–unaided differences, sensitivity to known acoustic transformations, or relationships with perceptual or clinical outcomes. The repeatability of aided recordings also remains to be established because nonlinear and adaptive hearing devices may respond differently across repeated presentations. The 37 descriptors should therefore be regarded as a development-derived analytical library rather than a definitive or statistically optimized feature set.

A second limitation concerns the external acquisition pathway. Electrical loopback testing showed no relevant spectral alteration in the cable–audio-interface–Praat segment, and repeated acoustic recordings demonstrated high within-path repeatability. However, frequency-dependent differences were observed between the spectra displayed by OTOsuite and those derived from the AURICAL FreeFit analog monitoring output. The two outputs were reproducible within their respective pathways but were not numerically interchangeable.

At the time of revision, sufficient technical documentation or formal clarification from Natus regarding the transfer characteristics and internal processing of the AURICAL FreeFit analog monitoring output was not available. Consequently, the precise origin of the observed cross-path differences could not be determined. The post-study stereo bridge showed that the discrepancy persisted with an alternative cable topology and therefore did not support the mono cable as its principal cause. Nevertheless, the additional tests indicated that cable configuration may influence the recorded frequency response. The mono TS connection used for all study recordings was therefore retained as the standardized study configuration. This choice ensures internal consistency but does not establish its superiority over other cable configurations.

The intended paired application uses the same ear, REM system, monitoring output, cable configuration, audio interface, gain settings, and analytical procedure for both unaided and aided recordings. This design improves internal consistency but does not demonstrate absolute spectral accuracy or guarantee transferability to other systems. Platform-specific verification is required before pooling or comparing results obtained with different REM platforms, monitoring outputs, cables, or audio interfaces. Future technical work should evaluate documented raw or compensated monitoring outputs, direct digital signal export, and additional REM systems.

Temporal alignment was performed by a single investigator, so inter-operator variability could not be estimated from the present data and the transferability of the procedure to other operators remains to be demonstrated. Within-operator variability is not a hidden source of error here, because independent alignment of the two sessions places it inside the reported test–retest differences. Future implementations should incorporate automated landmark detection or cross-correlation-based alignment and quantify any residual operator-related variability.

No formal a priori sample-size calculation or prespecified confidence-interval precision criterion was used for the test–retest study. The cohort comprised 25 participants, and the primary analysis included 24 right ears after exclusion of one right ear because of tympanic membrane perforation. Although the achieved 95% confidence intervals quantify the precision of the estimates, their width varied substantially across descriptors. The reliability estimates should therefore be interpreted as preliminary and require confirmation in a larger independent cohort.

The 25 contralateral left ears were analyzed separately as a within-sample sensitivity analysis. Because these ears were obtained from the same participants included in the primary analysis, this analysis examined the robustness of the principal pattern to the choice of ear but did not constitute an independent replication.

The Core, Candidate, and Secondary classification was exploratory and post hoc. The same primary right-ear dataset was used both to estimate descriptor reliability and to assign descriptors to tiers. Consequently, the apparent stability and generalizability of the tier classification cannot be established from the present data and must be evaluated in an independent sample.

The separate 26-ear Method-Development Dataset was not intended to establish population norms. All available ear recordings were retained because the analyses were descriptive and focused on variability across standardized ear-level recordings. Where bilateral recordings from the same participant were included, the observations were not fully independent at the participant level. The resulting distributions should therefore be regarded as preliminary technical references rather than independent estimates of intersubject variability, clinical reference intervals, or normative values.

Coefficients of variation were used solely as descriptive indicators. Although dimensionless, CVs are not directly comparable across descriptors representing different acoustic constructs and can be unstable or uninterpretable for signed variables, logarithmic measures, variables without a meaningful zero, or descriptors with means close to zero. In addition, some segment-dependent measurements were non-evaluable when the relevant recording interval contained insufficient signal energy. Larger datasets are needed to establish prespecified evaluability criteria and appropriate descriptor-specific measures of variability.

Twelve descriptors were extracted from two selected vowel tokens using automatic formant tracking without systematic manual verification. Tracking errors, local signal-energy differences, and the restricted vowel sample may have contributed to the variable reliability of some vowel-derived measures. Future studies should include prespecified tracking-quality criteria, blinded verification, and a broader vowel sample.

Finally, this study used one standardized Italian speech stimulus and a single REM platform. Recordings were obtained by multiple audiologists at independent clinical centers using the same acquisition protocol, but center- and operator-specific effects were not formally estimated. Generalizability to other languages, speakers, presentation levels, stimuli, and REM systems therefore remains to be established. Future within-ear aided–unaided studies should evaluate descriptor sensitivity and redundancy, aided-recording repeatability, and relationships with established acoustic and perceptual measures, including speech intelligibility, listening effort, perceived quality, and patient-reported benefit.

## 5. Conclusions

This study evaluated the stability and test–retest repeatability of the unaided intracanal recording used as the Individual Acoustic Reference in the SpettroREM method. The analysis included a 26-ear Method-Development Dataset for descriptive characterization of the acoustic descriptors and a test–retest cohort of 25 participants. The primary reliability analysis comprised 24 right ears after exclusion of one right ear because of tympanic membrane perforation; the 25 contralateral left ears were analyzed separately as a within-sample sensitivity analysis and were not pooled with the primary observations.

The results support repeatability principally for global spectral organization and selected additional descriptors. The main global spectral pattern was reproduced in the contralateral sensitivity analysis, but repeatability was not uniform across the complete descriptor library. Several segmental, vowel-derived, recording-level-dependent, and temporal-envelope descriptors showed greater uncertainty or were occasionally non-evaluable because of insufficient signal energy. The findings therefore do not establish the repeatability of every descriptor or of the IAR as a single multidimensional pattern.

Electrical testing supported the spectral transparency of the cable–audio interface–Praat segment under the tested settings, whereas repeated acoustic recordings demonstrated high within-path repeatability. However, frequency-dependent differences between the OTOsuite display and the external recording preclude treating the two outputs as numerically interchangeable and limit transferability across acquisition systems. Standardized use of the same REM system, monitoring output, mono TS cable, audio interface, gain settings, and analytical procedure supports internally consistent paired measurements but does not establish absolute spectral accuracy or cross-platform equivalence.

This study should therefore be interpreted as a preliminary reliability assessment of the unaided reference and its development-derived 37-descriptor library rather than as validation of the complete SpettroREM method. The exploratory Core, Candidate, and Secondary classification was derived post hoc from the same dataset used to estimate reliability and requires confirmation in an independent sample. Direct within-ear aided–unaided studies are required to establish sensitivity to device-induced transformations, aided-recording repeatability, clinical and perceptual relevance, and the most informative non-redundant descriptor subset.

## 6. Declaration of Generative AI and AI-Assisted Technologies

During the preparation of this work, the authors used generative AI tools (Claude (Anthropic) Opus 5.5, Anthropic, and ChatGPT (Opena AI), GPT-6.1, OpenAI) to assist with language editing, graphical preparation, and checks of internal textual and numerical consistency. The authors subsequently reviewed and verified all generated or revised content and take full responsibility for the final manuscript. The tools were not used to generate original experimental data or to make autonomous scientific decisions.

## Figures and Tables

**Table 1:** Functional organization and test–retest behavior of the 37 SpettroREM descriptors across six acoustic domains. For each domain, the table reports the number of descriptors, the principal acoustic information conveyed, and the overall methodological behavior observed in the stability and repeatability analyses. Detailed descriptor-level numerical results are reported in Supplementary Tables S2–S5.

Analytical Domain	No. of Descriptors	Principal Acoustic Information	Methodological Behavior in the Present Study
Global spectral organization	7	Long-term spectral distribution, spectral peak, centroid, spread, and energy across broad frequency bands	Showed the most consistent stability and test–retest repeatability. The principal pattern was reproduced in the separate contralateral analysis.
Fricative analysis	4	High-frequency energy and spectral characteristics of the selected /s/ segment	Repeatability varied among descriptors. Measures dependent on local level or signal energy were less consistent and require cautious interpretation.
Vowel acoustics	12	Formant structure, spectral centroid, intensity, and aperiodicity of the selected vowel segments	Showed heterogeneous repeatability. Results may be affected by local signal level, restricted vowel sampling, and automatic formant-tracking uncertainty.
Vowel geometry	4	Relative relationships and distances between the formant characteristics of the selected vowels	Repeatability varied across measures. Derived distances are mathematically related to their component formant variables and do not provide independent evidence.
Temporal envelope	4	Attack and post-vocalic decay characteristics	Showed heterogeneous repeatability. Some estimates were non-evaluable when the relevant segment contained insufficient signal energy.
Speech contrast	6	Within-speaker P90–P10 intensity ranges and local recording-level measures for selected speech events	Provided complementary information on level relationships, but repeatability was not uniform across descriptors. Clinical relevance and sensitivity to hearing-device processing remain to be established.

## Data Availability

The data that support the findings of this study are available from the corresponding author, S.B., upon reasonable request, subject to the terms of participant consent and applicable data-protection regulations.

## Supplementary Materials

The following supporting information can be downloaded at: https://www.mdpi.com/article/10.3390/audiolres16050158/s1, Table S1A: Distribution of exploratory measures, primary descriptors, and accessory outputs across the six SpettroREM analytical domains; Table S1: Developmental origin, acoustic information, extraction method, methodological relevance, and final classification of the 44 exploratory SpettroREM measures; Table S2: Descriptive statistics of the 37 acoustic descriptors in the Method-Development Dataset; Table S3: Exploratory quality-control limits derived from the Method-Development Dataset; Table S4: Test–retest reliability of the 37 acoustic descriptors in the quality-controlled primary right-ear Repeatability Dataset; Figure S1: Test–retest reliability of all 37 SpettroREM descriptors in the primary right-ear analysis; Table S5: Absolute test–retest agreement for the 37 acoustic descriptors; Table S6: Acquisition and analysis specifications of the SpettroREM implementation evaluated in the present study; Table S7: Descriptor-level test–retest reliability in the contralateral left-ear sensitivity analysis; Figure SB1: Technical validation workflow used to evaluate the external SpettroREM acquisition pathway; Table SA1: Validation components and pre-specified interpretation; Table SA2: Primary mono-cable acoustic quality control and repeatability; Table SA3: OTOsuite one-third-octave values from the primary right-ear acoustic validation (dB SPL); Table SA4: Mean-normalised external Praat spectra from the primary right-ear acoustic validation (dB); Table SA5: Primary cross-path spectral-shape agreement; Table SA6: Secondary stereo electrical bridge: mean relative transfer by band (dB); Table SA7: Secondary stereo acoustic bridge: repeatability and cross-path agreement; Table SA8: Interpretation matrix; Figure SB2: Primary mono-cable acoustic validation of the external SpettroREM acquisition pathway; Figure SB3: Mean relative transfer functions obtained in the post-study stereo bridge experiment. Figures SB1–SB3 and Tables SA1–SA8 belong to the Supplementary Technical Validation (Supplementary Methods), which also describes the procedural details of the validation of the external recording pathway.
