# Supplementary material for: Stability and Test–Retest Reliability of the Individual Acoustic Reference for Within-Subject Analysis of Hearing Device-Processed Speech: The SpettroREM Method
Source: Audiol Res. 2026 Oct 11;16(5):158. doi: 10.3390/audiolres16050158 (PMC13701301; doi:10.3390/audiolres16050158)
Supplement: Supplementary file 1 [file audiolres-16-00158-s001.zip › bozza editor aggiornata+supplementary/audiolres-4490445-supplementary.pdf]

Supplementary Materials

Stability and Test–Retest Reliability of the Individual Acoustic Reference for Within-Subject Analysis of Hearing Device–Processed Speech: The SpettroREM Method

S. Burdo [1,†], E. Carini [2], G. Impalà [3], and D. Brotto [3,4]

[1]Independent Researcher, Varese, Italy
[2]Institute for Maternal and Child Health–IRCCS Burlo Garofolo, 34137 Trieste, Italy
[3]UOC Otorinolaringoiatria/Otorhinolaryngology–Head and Neck Section, Azienda Ospedale-Università Padova, Via Giustiniani 2, 35128 Padova, Italy
[4]Department of Neurosciences, University of Padova, 35122 Padova, Italy
[†]Former Director of the Audiovestibology Unit, ASST dei Sette Laghi–Ospedale di Circolo di Varese, Varese, Italy

Manuscript ID: audiolres-4490445 (*Audiology Research*)

This file contains the Supplementary Material of the article: Supplementary Tables S1A and S1–S7, Supplementary Figure S1, and the Supplementary Technical Validation material, comprising Tables SA1–SA8 and Figures SB1–SB3. Abbreviations, dataset names, and the exploratory reliability tiers (Core, Candidate, and Secondary) are defined in the main article, and section numbers refer to the main text.

Supplementary Tables and Figure

Supplementary Table S1A. Distribution of exploratory measures, primary descriptors, and accessory outputs across the six SpettroREM analytical domains.

Domain	Exploratory measures	Primary descriptors	Accessory outputs
Global LTASS Organization	8	7	1
Fricative Spectral Characteristics	6	4	2
Vowel Acoustics	14	12	2
Vowel Geometry	6	4	2
Temporal-Envelope Dynamics	4	4	0
Speech-Level and Contrast Characteristics	6	6	0
Total	44	37	7

Supplementary Table S1. Developmental origin, acoustic information, extraction method, methodological relevance, and final classification of the 44 exploratory SpettroREM measures.

No.	Domain	Descriptor	Unit	Acoustic information	Extraction and developmental origin	Clinical/ methodological relevance	Final classification
1	Global LTASS Organization	LTASS peak level	dB	Level of the dominant component of the long-term speech spectrum	Extracted from the long-term spectrum of the complete recorded speech signal using Praat	Quantifies the magnitude of the dominant spectral component	Primary
2		LTASS peak frequency	Hz	Frequency at which the long-term speech spectrum reaches its maximum level	Extracted from the long-term spectrum of the complete recorded speech signal using Praat	Identifies the frequency region of maximum spectral emphasis	Primary
3		Global spectral centroid	Hz	Overall spectral center of gravity of the speech signal	Calculated from the long-term spectrum of the complete recorded speech signal	Characterizes the overall balance between lower- and higher-frequency speech energy	Primary
4		Spectral spread	Hz	Dispersion of spectral energy around the global centroid	Derived from the long-term spectrum of the complete recorded speech signal	Quantifies the overall concentration or dispersion of speech energy	Primary
5		LTASS 0–1 kHz	dB	Mean speech energy within the low-frequency band	Band-specific energy derived from the long-term spectrum of the complete recorded speech signal	Represents the low-frequency component of the speech spectrum	Primary
6		LTASS 1–4 kHz	dB	Mean speech energy within the middle-frequency band	Band-specific energy derived from the long-term spectrum of the complete recorded speech signal	Represents the region containing a substantial proportion of speech information	Primary
7		LTASS 4–8 kHz	dB	Mean speech energy within the high-frequency band	Band-specific energy derived from the long-term spectrum of the complete recorded speech signal	Represents the high-frequency component of the speech spectrum	Primary
8		LTASS HF/LF	dB	Balance between high- and low-frequency speech energy	Ratio derived from the LTASS frequency-band measures	Provides a synthetic measure of global spectral-energy redistribution	Accessory — derived from information already represented by the primary LTASS band measures

(Supplementary Table S1 – continued)

No.	Domain	Descriptor	Unit	Acoustic information	Extraction and developmental origin	Clinical/ methodological relevance	Final classification
9	Fricative Spectral Characteristics	/s/intensity	dB	Mean acoustic level of the selected fricative segment	Energy-mean intensity calculated in five overlapping 80-ms windows centred at −40, −20, 0, +20, and +40 ms relative to the predefined /s/ marker; final value obtained by averaging the valid estimates	Quantifies the local acoustic level of a weak high-frequency consonantal segment	Primary
10		/s/HF/LF	dB	Ratio between high- and low-frequency fricative energy	Calculated separately in the five overlapping windows and averaged across valid estimates	Characterizes preservation or redistribution of high-frequency consonantal energy	Primary
11		/s/HNR	dB	Harmonic-to-noise organization of the selected fricative segment	Praat-based HNR calculated separately in the five overlapping windows and averaged across valid estimates	Describes the regularity of the fricative signal relative to its noise-like component	Primary
12		/s/robust spectral centroid	Hz	Robust estimate of the location of fricative spectral energy	Energy-weighted centroid derived from predefined frequency bands in each of the five overlapping windows and averaged across valid estimates	Provides an alternative estimate of the spectral location of fricative energy	Accessory — partially overlaps with the primary fricative spectral-balance descriptors
13		/s/spectral slope	dB/ oct	Rate of change in fricative spectral energy as a function of frequency	Derived from the spectral distribution in each of the five overlapping windows and averaged across valid estimates	Characterizes the direction and steepness of fricative spectral-energy redistribution	Accessory — partially overlaps with /s/ HF/LF and spectral brightness
14		/s/spectral brightness	kHz	High-frequency emphasis within the fricative spectrum	Derived in each of the five overlapping windows and averaged across valid estimates	Provides a synthetic representation of the high-frequency distribution of fricative energy	Primary
15	Vowel Acoustics	V1 F1	Hz	First-formant frequency of the first selected vowel segment	Praat formant analysis in five overlapping 80-ms windows; mean of the valid estimates	Characterizes the low-frequency resonance of V1	Primary

(Supplementary Table S1 – continued)

No.	Domain	Descriptor	Unit	Acoustic information	Extraction and developmental origin	Clinical/ methodological relevance	Final classification
16		V1 F2	Hz	Second-formant frequency of the first selected vowel segment	Praat formant analysis in five overlapping 80-ms windows; mean of the valid estimates	Characterizes the middle-frequency spectral organization of V1	Primary
17		V1 F3	Hz	Third-formant frequency of the first selected vowel segment	Praat formant analysis in five overlapping 80-ms windows; mean of the valid estimates	Characterizes the higher-frequency resonance structure of V1	Primary
18		V1 intensity	dB	Mean acoustic level of the first selected vowel segment	Energy-mean intensity calculated in five overlapping 80-ms windows and averaged across valid estimates	Quantifies the local acoustic level of V1	Primary
19		V1 HNR	dB	Harmonic-to-noise organization of the first selected vowel segment	Praat-based HNR calculated in five overlapping 80-ms windows and averaged across valid estimates	Provides a measure of harmonic organization within V1	Accessory — partially overlaps with the representation of vowel periodicity provided by V1 aperiodicity
20		V1 aperiodicity	index	Departure from periodic organization within the first selected vowel segment	Calculated in five overlapping 80-ms windows and averaged across valid estimates	Characterizes irregularity of the harmonic structure of V1	Primary
21		V1 spectral centroid	Hz	Spectral center of gravity of the first selected vowel segment	Calculated in five overlapping 80-ms windows and averaged across valid estimates	Characterizes the overall spectral distribution of V1	Primary
22		V2 F1	Hz	First-formant frequency of the second selected vowel segment	Praat formant analysis in five overlapping 80-ms windows; mean of the valid estimates	Characterizes the low-frequency resonance of V2	Primary
23		V2 F2	Hz	Second-formant frequency of the second selected vowel segment	Praat formant analysis in five overlapping 80-ms windows; mean of the valid estimates	Characterizes the middle-frequency spectral organization of V2	Primary
24		V2 F3	Hz	Third-formant frequency of the second selected vowel segment	Praat formant analysis in five overlapping 80-ms windows; mean of the valid estimates	Characterizes the higher-frequency resonance structure of V2	Primary

(Supplementary Table S1 – continued)

No.	Domain	Descriptor	Unit	Acoustic information	Extraction and developmental origin	Clinical/ methodological relevance	Final classification
25		V2 intensity	dB	Mean acoustic level of the second selected vowel segment	Energy-mean intensity calculated in five overlapping 80-ms windows and averaged across valid estimates	Quantifies the local acoustic level of V2	Primary
26		V2 HNR	dB	Harmonic-to-noise organization of the second selected vowel segment	Praat-based HNR calculated in five overlapping 80-ms windows and averaged across valid estimates	Provides a measure of harmonic organization within V2	Accessory — partially overlaps with the representation of vowel periodicity provided by V2 aperiodicity
27		V2 aperiodicity	index	Departure from periodic organization within the second selected vowel segment	Calculated in five overlapping 80-ms windows and averaged across valid estimates	Characterizes irregularity of the harmonic structure of V2	Primary
28		V2 spectral centroid	Hz	Spectral center of gravity of the second selected vowel segment	Calculated in five overlapping 80-ms windows and averaged across valid estimates	Characterizes the overall spectral distribution of V2	Primary
29	Vowel Geometry	ΔF1 (V2−V1)	Hz	Difference in F1 between the two selected vowel segments	Derived from the window-averaged F1 values of V1 and V2	Characterizes the relative positioning of V1 and V2 along the F1 dimension	Primary
30		ΔF2 (V2−V1)	Hz	Difference in F2 between the two selected vowel segments	Derived from the window-averaged F2 values of V1 and V2	Characterizes the relative positioning of V1 and V2 along the F2 dimension	Primary
31		Vowel distance F1–F2	Hz	Euclidean separation between V1 and V2 in the F1–F2 plane	Geometrical distance derived from the window-averaged F1 and F2 values	Quantifies preservation or compression of the two-vowel configuration in formant space	Primary
32		Δcentroid (V2−V1)	Hz	Difference in spectral centroid between V1 and V2	Derived from the window-averaged spectral centroids of V1 and V2	Describes the relative displacement of the overall spectral distributions of the two vowels	Accessory — partially overlaps with the two primary vowel-centroid descriptors
33		Extended vowel distance	Hz	Multidimensional separation between V1 and V2	Derived from multiple window-averaged vowel measurements	Provides an extended representation of inter-vowel spectral separation	Accessory — partially overlaps with the primary vowel-geometrical distance measures

(Supplementary Table S1 – continued)

No.	Domain	Descriptor	Unit	Acoustic information	Extraction and developmental origin	Clinical/ methodological relevance	Final classification
34		Normalized distance z	z	Standardized multidimensional separation between V1 and V2	Calculated from ΔF1, ΔF2, and Δcentroid after scaling by their predefined reference values	Permits comparison of inter-vowel separation with reduced dependence on the original measurement scales	Primary
35	Temporal-Envelope Dynamics	/s/attack 10–90%	ms	Time required for the fricative envelope to rise from 10% to 90% of its local amplitude range	Calculated from the continuous intensity contour within the predefined /s/ envelope interval	Characterizes the temporal onset of the fricative segment	Primary
36		V1 attack 10–90%	ms	Time required for the vowel envelope to rise from 10% to 90% of its local amplitude range	Calculated from the continuous intensity contour within the predefined V1 envelope interval	Characterizes the temporal onset of the first vowel segment	Primary
37		T−3 dB post-vowel decay	ms	Time required for the post-vocalic envelope to decrease by 3 dB	Calculated from the continuous intensity contour within the predefined post-vowel decay interval	Characterizes the early phase of post-vocalic signal decay	Primary
38		T−6 dB post-vowel decay	ms	Time required for the post-vocalic envelope to decrease by 6 dB	Calculated from the continuous intensity contour within the predefined post-vowel decay interval	Characterizes a more extended phase of post-vocalic signal decay	Primary
39	Speech-Level and Contrast Characteristics	Female-speaker P90–P10	dB	Robust within-speaker intensity range of the female speech interval	Difference between the 90th and 10th percentiles of the intensity distribution calculated over the predefined female-speaker interval	Characterizes preservation or modification of intensity variation within female speech and permits speaker-specific analysis without pooling acoustically and prosodically different speech intervals	Primary

(Supplementary Table S1 – continued)

No.	Domain	Descriptor	Unit	Acoustic information	Extraction and developmental origin	Clinical/ methodological relevance	Final classification
40		Male-speaker P90–P10	dB	Robust within-speaker intensity range of the male speech interval	Difference between the 90th and 10th percentiles of the intensity distribution calculated over the predefined male-speaker interval	Characterizes preservation or modification of intensity variation within male speech and permits speaker-specific analysis without pooling acoustically and prosodically different speech intervals	Primary
41		Child-speaker P90–P10	dB	Robust within-speaker intensity range of the child speech interval	Difference between the 90th and 10th percentiles of the intensity distribution calculated over the predefined child-speaker interval	Characterizes preservation or modification of intensity variation within child speech and permits separate analysis of a speech interval with distinct level, spectral, and prosodic characteristics	Primary
42		/s/ local intensity	dB	Absolute local level of an additional consonantal event within the continuous speech stimulus	Energy-mean intensity calculated over one dedicated 80-ms window centred on an /s/ event distinct from the /s/ used for the five-window fricative analysis	Provides a local consonantal-level reference; it is not itself a contrast measure	Primary
43		Vowel1 local intensity	dB	Absolute local level of an additional vowel event within the continuous speech stimulus	Energy-mean intensity calculated over one dedicated 80-ms window centred on a vowel event distinct from V1 used for the five-window vowel analysis	Provides a first local vowel-level reference; it is not itself a contrast measure	Primary

44	Vowel2 local intensity	dB	Absolute local level of a second additional vowel event within the continuous speech stimulus	Energy-mean intensity calculated over one dedicated 80-ms window centred on a vowel event distinct from V2 used for the five-window vowel analysis	Provides a second local vowel-level reference; it is not itself a contrast measure	Primary

Complete list of the 44 acoustic measures implemented during the exploratory development of the SpettroREM Method. For each measure, the table reports its analytical domain, name, measurement unit, acoustic information, extraction and developmental origin, principal clinical or methodological relevance, and final classification. The exploratory library comprised 37 primary IAR descriptors and seven accessory outputs retained for complementary analyses. The measures are organized into six analytical domains and collectively provide a multidimensional, non-normative physical description of the real-ear speech signal. They are intended to be interpreted as complementary characteristics of the recorded signal rather than as statistically independent outcome measures. Classification as a primary descriptor or accessory output was based on conceptual role, derivability, and partial informational overlap and was not based on retrospective exclusion of measures showing unfavourable test–retest reliability. Female-, male-, and child-speaker P90–P10 values were calculated separately to preserve speaker-specific information and avoid concealing potential differences across acoustically and prosodically distinct speech intervals through averaging. The /s/, V1, and V2 local-intensity measures represent local recording-level measurements and are not themselves measures of speech contrast or independently calibrated SPL values. The standardized inter-vowel distance uses predefined scaling constants rather than sample-derived standard deviations and does not constitute statistical standardization against a normative population. "Index" and "z" denote dimensionless quantities. Abbreviations: LTASS = Long-Term Average Speech Spectrum; HNR = Harmonic-to-Noise Ratio; HF/LF = High-to-Low Frequency energy ratio; P90–P10 = difference between the 90th and 10th percentiles of the intensity distribution; F1–F3 = first, second, and third formants; V1 and V2 = first and second analysed vowels; ΔF1 and ΔF2 = between-vowel formant differences (V2−V1); Δcentroid = difference in spectral centroid between V2 and V1; /s/ = analysed voiceless alveolar fricative.

Supplementary Table S2. Descriptive statistics of the 37 acoustic descriptors in the Method-Development Dataset (n = 26 unaided ear recordings, except where noted). For each descriptor the table reports the number of evaluable recordings, minimum, first quartile (Q1), median, third quartile (Q3), maximum, mean, and standard deviation. One recording per ear was retained; where bilateral recordings from the same participant were available, both ears were treated as separate ear-level observations, and the analyses did not assume complete independence at the participant level. Values are descriptive, preliminary, and non-normative, and should not be interpreted as normative values, diagnostic thresholds, or clinical reference intervals. A dash in the Unit column denotes a dimensionless quantity.

**Domain**	**Descriptor**	**Unit**	**n**	**Min**	**Q1**	**Median**	**Q3**	**Max**	**Mean**	**SD**
Envelope	/s/ attack 10–90%	ms	26	20.00	68.25	74.00	79.75	110.00	73.00	15.85
	T–3 dB post-vowel decay	ms	25	23.00	55.00	63.00	81.00	120.00	69.52	22.61
	T–6 dB post-vowel decay	ms	24	43.00	86.25	108.50	143.25	646.00	168.67	162.53
	V1 attack 10–90%	ms	26	26.00	48.00	59.50	78.50	171.00	66.77	32.55
Fricative	HF/LF	dB	26	−21.87	−14.90	−12.12	−8.04	1.78	−11.42	5.76
	HNR	dB	26	−3.70	−2.60	−2.27	−2.01	−0.63	−2.22	0.74
	Intensity	dB	26	28.76	33.37	36.43	39.81	46.48	36.45	4.59
	Spectral brightness	kHz	26	3.07	3.17	3.36	3.56	4.24	3.41	0.32
LTASS	Global spectral centroid	Hz	26	804.00	953.75	1211.00	1351.25	1900.00	1220.65	306.53
	LTASS peak frequency	Hz	26	350.00	350.00	400.00	550.00	2050.00	623.08	499.25
	LTASS peak level	dB	26	15.49	19.74	23.06	24.32	30.69	22.54	3.78
	LTASS 0–1 kHz	dB	26	9.91	14.11	17.46	18.24	25.11	16.84	3.55
	LTASS 1–4 kHz	dB	26	2.08	7.29	9.16	12.57	18.88	9.81	4.56
	LTASS 4–8 kHz	dB	26	−24.93	−19.42	−15.86	−11.97	−1.72	−15.62	5.52
	Spectral spread	Hz	26	688.00	810.50	910.50	980.50	1427.00	925.96	156.88
Speech-Level and Contrast	/s/ local intensity	dB	26	45.50	51.00	54.34	56.22	61.97	53.81	3.84
	Child contrast	dB	26	8.41	9.20	9.88	10.37	11.93	9.90	0.96
	Female contrast	dB	26	8.43	9.94	10.68	11.26	12.54	10.64	0.93
	Male contrast	dB	26	4.74	5.21	5.66	6.09	7.22	5.69	0.60
	Vowel1 local intensity	dB	26	48.76	54.18	56.51	58.43	64.70	56.49	4.11
	Vowel2 local intensity	dB	26	53.80	58.15	60.43	63.98	68.93	60.76	4.11
Vowels	V1 F1	Hz	26	482.00	624.50	715.50	772.50	1105.00	719.35	140.10
	V1 F2	Hz	26	1520.00	1718.50	1820.50	1913.00	2062.00	1811.96	128.58
	V1 F3	Hz	26	2250.00	2458.00	2569.00	2611.75	3011.00	2534.19	154.59
	V1 aperiodicity	—	26	0.26	0.37	0.42	0.48	0.54	0.42	0.07
	V1 centroid	Hz	26	569.00	864.25	1254.00	1446.75	1791.00	1185.69	360.72
	V1 intensity	dB	26	48.67	51.76	54.85	56.19	62.66	54.40	3.88
	V2 F1	Hz	26	766.00	862.25	898.50	954.50	1102.00	902.50	79.07
	V2 F2	Hz	26	1275.00	1559.50	1610.00	1695.25	1919.00	1643.42	145.66
	V2 F3	Hz	26	2295.00	2493.25	2635.50	2671.50	3009.00	2610.08	155.72
	V2 aperiodicity	—	26	0.39	0.47	0.51	0.54	0.64	0.51	0.06
	V2 centroid	Hz	26	970.00	1200.75	1384.50	1508.50	2005.00	1404.54	247.81
	V2 intensity	dB	26	48.43	52.65	55.07	57.53	64.21	55.24	3.94
Vowel Geometry	ΔF1 (V2–V1)	Hz	26	−146.00	129.08	181.45	257.78	423.00	183.22	113.92
	ΔF2 (V2–V1)	Hz	26	−331.40	−237.50	−160.85	−105.30	−31.70	−168.41	87.99
	Standardized inter-vowel distance	—	26	0.92	1.77	2.12	2.66	4.33	2.24	0.81
	Vowel distance F1–F2	Hz	26	156.60	202.73	274.55	325.28	453.30	274.95	80.68

Note: T−3 dB and T−6 dB post-vowel decay were evaluable in 25 and 24 recordings, respectively, owing to insufficient signal energy in the corresponding segments.

Supplementary Table S3. Exploratory quality-control limits derived from the Method-Development Dataset ($n = 26$). For each descriptor the table reports the number of evaluable recordings, first quartile (Q1), third quartile (Q3), interquartile range (IQR), and the Tukey lower and upper fences (Q1−1.5×IQR and Q3+1.5×IQR). These are exploratory acquisition-screening limits, not clinical reference intervals, diagnostic thresholds, or automatic exclusion criteria. They are intended only to flag recordings that may require technical inspection or repetition and were not used retrospectively to exclude recordings or descriptors from the present analyses. Because several descriptors are correlated, the occurrence of multiple deviations should not be interpreted using a binomial model that assumes statistical independence.

**Domain**	**Descriptor**	**Unit**	**n**	**Q1**	**Q3**	**IQR**	**Lower limit**	**Upper limit**
Envelope	/s/ attack 10–90%	ms	26	68.25	79.75	11.50	51.00	97.00
	T–3 dB post-vowel decay	ms	25	55.00	81.00	26.00	16.00	120.00
	T–6 dB post-vowel decay	ms	24	86.25	143.25	57.00	0.75	228.75
	V1 attack 10–90%	ms	26	48.00	78.50	30.50	2.25	124.25
Fricative	HF/LF	dB	26	−14.90	−8.04	6.86	−25.19	2.24
	HNR	dB	26	−2.60	−2.01	0.59	−3.49	−1.12
	Intensity	dB	26	33.37	39.81	6.44	23.71	49.47
	Spectral brightness	kHz	26	3.17	3.56	0.39	2.58	4.15
LTASS	Global spectral centroid	Hz	26	953.75	1351.25	397.50	357.50	1947.50
	LTASS peak frequency	Hz	26	350.00	550.00	200.00	50.00	850.00
	LTASS peak level	dB	26	19.74	24.32	4.58	12.87	31.19
	LTASS 0–1 kHz	dB	26	14.11	18.24	4.13	7.92	24.43
	LTASS 1–4 kHz	dB	26	7.29	12.57	5.29	−0.64	20.50
	LTASS 4–8 kHz	dB	26	−19.42	−11.97	7.45	−30.58	−0.80
	Spectral spread	Hz	26	810.50	980.50	170.00	555.50	1235.50
Speech-Level and Contrast	/s/ local intensity	dB	26	51.00	56.22	5.22	43.16	64.06
	Child contrast	dB	26	9.20	10.37	1.17	7.45	12.12
	Female contrast	dB	26	9.94	11.26	1.32	7.97	13.23
	Male contrast	dB	26	5.21	6.09	0.87	3.90	7.39
	Vowel1 local intensity	dB	26	54.18	58.43	4.25	47.81	64.80
	Vowel2 local intensity	dB	26	58.15	63.98	5.83	49.41	72.72
Vowels	V1 F1	Hz	26	624.50	772.50	148.00	402.50	994.50
	V1 F2	Hz	26	1718.50	1913.00	194.50	1426.75	2204.75
	V1 F3	Hz	26	2458.00	2611.75	153.75	2227.38	2842.38
	V1 aperiodicity	—	26	0.37	0.48	0.12	0.19	0.66
	V1 centroid	Hz	26	864.25	1446.75	582.50	0.00	2320.50
	V1 intensity	dB	26	51.76	56.19	4.44	45.10	62.85
	V2 F1	Hz	26	862.25	954.50	92.25	723.88	1092.88
	V2 F2	Hz	26	1559.50	1695.25	135.75	1355.88	1898.88
	V2 F3	Hz	26	2493.25	2671.50	178.25	2225.88	2938.88
	V2 aperiodicity	—	26	0.47	0.54	0.07	0.36	0.65
	V2 centroid	Hz	26	1200.75	1508.50	307.75	739.13	1970.13
	V2 intensity	dB	26	52.65	57.53	4.88	45.32	64.85
Vowel Geometry	ΔF1 (V2–V1)	Hz	26	129.08	257.78	128.70	−63.98	450.83
	ΔF2 (V2–V1)	Hz	26	−237.50	−105.30	132.20	−435.80	93.00
	Standardized inter-vowel distance	—	26	1.77	2.66	0.89	0.43	3.99
	Vowel distance F1–F2	Hz	26	202.73	325.28	122.55	18.90	509.10

Note: For descriptors physically bounded at zero, negative lower fences were truncated at zero; this truncation was not applied to signed descriptors.

Supplementary Table S4. Test–retest reliability of the 37 acoustic descriptors in the quality-controlled primary right-ear Repeatability Dataset ($n = 24$ pairs, except where noted). The table reports the number of evaluable pairs, exploratory reliability tier, Pearson's r with its 95% confidence interval, ICC(A,1) with its 95% confidence interval, and a descriptive reliability label (after Koo and Li, 2016). ICC confidence intervals were estimated using 10,000 participant-level bootstrap resamples. Tier assignment was exploratory and post hoc.

**Domain**	**Descriptor**	**n**	**Tier**	**Pearson r**	**95% CI**		**ICC(A,1)**	**95% CI**		**Interpretation**
					low	*high*		*low*	*high*	
Envelope	/s/ attack 10–90%	24	Secondary	0.66	0.36	0.84	0.66	0.11	0.92	Moderate
	T–3 dB post-vowel decay	22	Secondary	0.22	−0.22	0.59	0.23	−0.18	0.71	Poor
	T–6 dB post-vowel decay	21	Secondary	0.56	0.16	0.80	0.54	0.18	0.84	Moderate
	V1 attack 10–90%	23	Secondary	0.58	0.21	0.80	0.57	0.12	0.89	Moderate
Fricative	HF/LF	24	Candidate	0.84	0.66	0.93	0.84	0.65	0.95	Good
	HNR	24	Secondary	0.43	0.04	0.71	0.41	0.22	0.79	Poor
	Intensity	24	Secondary	0.14	−0.28	0.52	0.14	−0.13	0.47	Poor
	Spectral brightness	24	Candidate	0.89	0.76	0.95	0.88	0.68	0.95	Good
LTASS	Global spectral centroid	24	Core	0.91	0.79	0.96	0.91	0.81	0.95	Excellent
	LTASS peak frequency	24	Core	0.91	0.81	0.96	0.91	0.76	1.00	Excellent
	LTASS peak level	24	Core	0.95	0.89	0.98	0.95	0.77	0.99	Excellent
	LTASS 0–1 kHz	24	Core	0.98	0.96	0.99	0.98	0.93	0.99	Excellent
	LTASS 1–4 kHz	24	Core	0.96	0.91	0.98	0.96	0.90	0.98	Excellent
	LTASS 4–8 kHz	24	Core	0.95	0.89	0.98	0.95	0.87	0.98	Excellent
	Spectral spread	24	Core	0.91	0.80	0.96	0.91	0.78	0.96	Excellent
Speech-Level and Contrast	/s/ local intensity	24	Secondary	0.23	−0.19	0.58	0.23	−0.17	0.55	Poor
	Child contrast	23	Secondary	0.73	0.45	0.88	0.64	0.45	0.83	Moderate
	Female contrast	24	Candidate	0.81	0.60	0.91	0.76	0.63	0.89	Good
	Male contrast	24	Secondary	0.11	−0.31	0.49	0.09	−0.14	0.74	Poor
	Vowel1 local intensity	24	Secondary	0.97	0.93	0.99	0.97	0.92	0.99	Excellent
	Vowel2 local intensity	24	Secondary	0.71	0.44	0.87	0.72	0.47	0.91	Moderate
Vowels	V1 F1	24	Secondary	0.54	0.17	0.77	0.53	−0.08	0.91	Moderate
	V1 F2	24	Secondary	0.67	0.36	0.84	0.67	0.15	0.88	Moderate
	V1 F3	24	Secondary	0.63	0.30	0.82	0.62	0.30	0.83	Moderate
	V1 aperiodicity	24	Secondary	0.66	0.35	0.84	0.67	0.25	0.87	Moderate
	V1 centroid	24	Candidate	0.76	0.51	0.89	0.76	0.55	0.91	Good
	V1 intensity	24	Secondary	0.94	0.87	0.97	0.94	0.83	0.98	Excellent
	V2 F1	24	Secondary	0.68	0.39	0.85	0.67	0.30	0.81	Moderate
	V2 F2	24	Secondary	0.35	−0.06	0.66	0.32	0.04	0.51	Poor
	V2 F3	24	Secondary	0.50	0.13	0.75	0.50	0.06	0.76	Moderate
	V2 aperiodicity	24	Secondary	0.22	−0.20	0.57	0.22	−0.23	0.61	Poor
	V2 centroid	24	Secondary	0.65	0.34	0.84	0.65	−0.05	0.86	Moderate
	V2 intensity	24	Secondary	0.35	−0.06	0.66	0.33	0.11	0.55	Poor
Vowel Geometry	Standardized inter-vowel distance	24	Secondary	0.49	0.11	0.75	0.49	−0.10	0.75	Poor
	Vowel distance F1–F2	24	Secondary	0.27	−0.15	0.60	0.26	−0.10	0.55	Poor
	ΔF1 (V2–V1)	24	Secondary	0.60	0.25	0.81	0.59	−0.31	0.90	Moderate
	ΔF2 (V2–V1)	24	Secondary	0.42	0.02	0.71	0.41	−0.02	0.67	Poor

Note: V1 attack 10–90% and child contrast were evaluable in 23 pairs, T−3 dB post-vowel decay in 22, and T−6 dB post-vowel decay in 21, owing to insufficient segment energy. Tiers are exploratory and do not constitute validated feature selection.

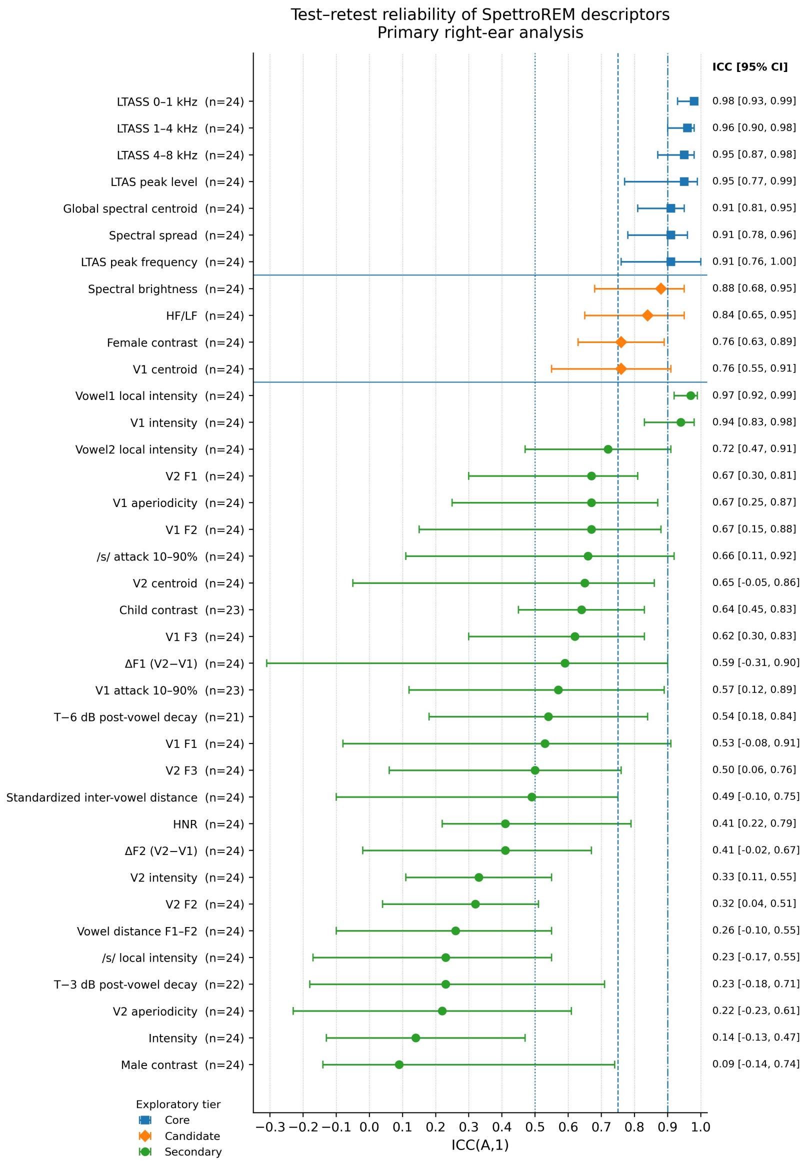

Points are ICC(A,1) estimates; error bars are 95% subject-level bootstrap confidence intervals (10,000 resamples). Vertical reference lines: 0.50, 0.75, and 0.90. Tier classification is exploratory and post hoc.

Supplementary Figure S1. Test–retest reliability of all 37 SpettroREM descriptors in the primary right-ear analysis. Points represent ICC(A,1) estimates, and error bars represent 95% participant-level bootstrap confidence intervals based on 10,000 resamples. Vertical reference lines indicate ICC values of 0.50, 0.75, and 0.90. Colours identify the exploratory tiers: blue squares indicate Core descriptors, orange diamonds indicate Candidate descriptors, and green circles indicate Secondary descriptors. The two horizontal blue lines separate the Core, Candidate, and Secondary sections of the plot. Tier assignment was hierarchical, exploratory, and post hoc. Secondary included descriptors with ICC <0.75 and directly recording-level-dependent local intensity measures, regardless of their ICC point estimates. Consequently, some Secondary descriptors show high ICC point estimates but were not classified as Core or Candidate. Numerical results are reported in Supplementary Table S4.

Supplementary Table S5. Absolute test–retest agreement for the 37 acoustic descriptors ($n = 24$ pairs, except where noted), from the quality-controlled Repeatability Dataset. The table reports the Mean Absolute Difference (MAD), Bland–Altman bias with its 95% confidence interval, the 95% limits of agreement (Lower LoA, Upper LoA), and the Repeatability Coefficient (RC). Differences are Session 2 minus Session 1.

**Domain**	**Descriptor**	**Unit**	**n**	**MAD**	**Bias**	**95% CI**		**Lower LoA**	**Upper LoA**	**RC**
						low	*high*			
Envelope	/s/ attack 10–90%	ms	24	9.29	−3.13	−9.10	2.85	−30.85	24.60	27.73
	T–3 dB post-vowel decay	ms	22	83.14	−10.50	−77.76	56.76	−307.84	286.84	297.34
	T–6 dB post-vowel decay	ms	21	79.71	39.62	−16.88	96.12	−203.64	282.88	243.26
	V1 attack 10–90%	ms	23	14.74	−5.96	−17.88	5.97	−60.02	48.10	54.06
Fricative	HF/LF	dB	24	2.61	−0.72	−2.28	0.84	−7.96	6.53	7.25
	HNR	dB	24	0.95	0.00	−0.68	0.69	−3.17	3.18	3.17
	Intensity	dB	24	3.46	0.25	−1.89	2.38	−9.65	10.14	9.90
	Spectral brightness	kHz	24	0.12	−0.04	−0.11	0.02	−0.35	0.26	0.31
LTASS	Global spectral centroid	Hz	24	51.55	−6.44	−32.95	20.08	−129.52	116.64	123.08
	LTASS peak frequency	Hz	24	12.50	−4.17	−19.31	10.98	−74.46	66.13	70.29
	LTASS peak level	dB	24	0.55	−0.23	−0.57	0.11	−1.80	1.34	1.57
	LTASS 0–1 kHz	dB	24	0.31	−0.11	−0.29	0.07	−0.95	0.72	0.83
	LTASS 1–4 kHz	dB	24	0.50	−0.05	−0.32	0.22	−1.30	1.20	1.25
	LTASS 4–8 kHz	dB	24	0.88	−0.40	−0.87	0.08	−2.61	1.82	2.21
	Spectral spread	Hz	24	36.96	−10.90	−30.23	8.43	−100.61	78.81	89.71
Speech-Level and Contrast	/s/ local intensity	dB	24	4.80	0.52	−2.13	3.17	−11.77	12.82	12.29
	Child contrast	dB	23	0.85	0.38	−0.18	0.94	−2.14	2.90	2.52
	Female contrast	dB	24	0.51	−0.04	−0.36	0.28	−1.52	1.43	1.48
	Male contrast	dB	24	0.66	0.21	−0.33	0.75	−2.30	2.73	2.52
	Vowel1 local intensity	dB	24	0.46	−0.07	−0.30	0.17	−1.16	1.02	1.09
	Vowel2 local intensity	dB	24	1.36	0.39	−0.60	1.38	−4.20	4.99	4.59
Vowels	V1 F1	Hz	24	31.22	13.51	−9.28	36.30	−92.27	119.28	105.77
	V1 F2	Hz	24	64.45	10.30	−29.56	50.16	−174.71	195.31	185.01
	V1 F3	Hz	24	83.18	26.32	−22.31	74.95	−199.41	252.04	225.72
	V1 aperiodicity	—	24	0.03	0.01	−0.01	0.02	−0.07	0.08	0.08
	V1 centroid	Hz	24	93.32	−9.51	−70.06	51.04	−290.55	271.54	281.05
	V1 intensity	dB	24	0.58	−0.02	−0.32	0.29	−1.43	1.39	1.41
	V2 F1	Hz	24	47.89	−1.90	−26.32	22.51	−115.23	111.42	113.32
	V2 F2	Hz	24	74.01	−21.94	−58.41	14.53	−191.23	147.35	169.29
	V2 F3	Hz	24	123.86	−12.27	−79.19	54.65	−322.89	298.35	310.62
	V2 aperiodicity	—	24	0.06	−0.02	−0.06	0.02	−0.20	0.16	0.18
	V2 centroid	Hz	24	110.76	−26.22	−84.51	32.07	−296.79	244.35	270.57
	V2 intensity	dB	24	2.92	0.99	−0.60	2.57	−6.37	8.34	7.35
Vowel Geometry	Standardized inter-vowel distance	—	24	0.41	0.12	−0.09	0.32	−0.84	1.08	0.96
	Vowel distance F1–F2	Hz	24	72.18	23.39	−15.59	62.36	−157.51	204.28	180.90
	ΔF1 (V2–V1)	Hz	24	54.20	−15.38	−49.44	18.67	−173.47	142.70	158.08
	ΔF2 (V2–V1)	Hz	24	105.25	−32.22	−82.80	18.36	−267.01	202.57	234.79

Note: Bias confidence intervals use the t distribution; limits of agreement are bias ±1.96 SD; RC is 1.96×SD of the differences.

Supplementary Table S6. Acquisition and analysis specifications of the SpettroREM implementation evaluated in the present study. The table summarizes the REM system, external recording pathway, recording parameters, standardized Italian speech stimulus, multicentre acquisition procedures, temporal-alignment method, quality-control procedure, and automated descriptor extraction used across the study recordings. Common acquisition and analysis settings were applied across participating centres and operators to minimize procedural variability. However, centre- and operator-specific effects were not estimated separately, and equivalent performance should not be assumed for other REM systems, monitoring outputs, cable configurations, audio interfaces, or acquisition settings without platform-specific technical verification.

**Parameter**	**Specification**
Acoustic REM system	AURICAL FreeFit/OTOsuite (Natus)
Probe microphone	Standard FreeFit probe microphone with calibrated probe tube
Signal output	AURICAL FreeFit analogue monitoring output
Connection cable	3.5-mm-to-6.35-mm mono TS cable; unchanged throughout the primary study
Audio interface	Focusrite Scarlett Solo (4th generation)
Input gain	Fixed across all study recordings
Recording format	WAV, mono, 44.1 kHz, 16-bit
Automatic processing	No automatic gain control, filtering, signal-level normalization, or post-recording equalization before descriptor extraction
Speech stimulus	Standardized Italian dialogue containing background noise, OTOsuite speech library no. 31
Presentation level	65 dB SPL at 1 m
Stimulus duration	Approximately 44 s
Additional recording time	Less than 1 min, excluding routine probe placement and calibration
Recording centres	Multiple participating clinical centres
Probe calibration	Performed before each measurement session or recording series according to the manufacturer's procedure
Operators	Multiple audiologists following the same standardized acquisition protocol
Analysis software	Praat version 6.4.25 with a dedicated SpettroREM script
Analysis mode	Offline
Temporal alignment	Operator identification of a predefined intensity landmark, followed by standardized temporal shifting and truncation to the common analysis interval
Descriptor extraction	Automated after temporal alignment and technical quality-control review
Number of descriptors	37
Analytical domains	6
Output	CSV file containing all descriptor values
Within-subject comparison	Intended paired comparison between aided and unaided recordings obtained from the same ear
Quality control	Performed before descriptor-level statistical analysis
Standardization across sites	Common acquisition and analysis settings applied across centres and operators; centre- and operator-specific effects were not estimated separately

Supplementary Table S7. Descriptor-level test–retest reliability in the contralateral left-ear sensitivity analysis. Statistics are based on 25 contralateral left-ear test–retest pairs from the same participants included in the primary analysis, except where insufficient segment energy reduced the number of evaluable pairs. Pearson confidence intervals use Fisher's z transformation; ICC(A,1) confidence intervals use 10,000 participant-level bootstrap resamples. This is a within-sample sensitivity analysis, not an independent replication, and no new reliability tiers were assigned.

**Domain**	**Descriptor**	**n**	**Pearson** r	**95% CI**		**ICC(A,1)**	**95% CI**	
				low	*high*		*low*	*high*
Envelope	/s/ attack 10–90%	24	0.63	0.30	0.82	0.58	0.20	0.83
	T–3 dB post-vowel decay	22	0.87	0.71	0.95	0.84	0.07	0.98
	T–6 dB post-vowel decay	21	0.05	−0.39	0.47	0.05	−0.35	0.45
	V1 attack 10–90%	25	0.89	0.75	0.95	0.89	0.27	0.99
Fricative	HF/LF	25	0.71	0.43	0.86	0.68	0.25	0.90
	HNR	25	0.36	−0.04	0.66	0.34	−0.12	0.61
	Intensity	25	−0.05	−0.44	0.35	−0.09	−0.39	0.25
	Spectral brightness	25	0.78	0.56	0.90	0.75	0.50	0.89
LTASS	Global spectral centroid	25	0.75	0.51	0.88	0.75	0.54	0.88
	LTASS peak frequency	25	−0.61	−0.81	−0.28	−0.18	−0.57	0.05
	LTASS peak level	25	0.93	0.84	0.97	0.93	0.80	0.97
	LTASS 0–1 kHz	25	0.91	0.81	0.96	0.91	0.68	0.97
	LTASS 1–4 kHz	25	0.84	0.67	0.93	0.84	0.65	0.93
	LTASS 4–8 kHz	25	0.94	0.86	0.97	0.94	0.87	0.97
	Spectral spread	25	0.89	0.76	0.95	0.89	0.80	0.94
Speech-Level and Contrast	/s/ local intensity	25	−0.11	−0.48	0.30	−0.09	−0.39	0.25
	Child contrast	25	0.83	0.65	0.92	0.82	0.72	0.87
	Female contrast	25	0.83	0.64	0.92	0.82	0.62	0.94
	Male contrast	25	0.79	0.57	0.90	0.79	0.47	0.91
	Vowel1 local intensity	25	0.86	0.70	0.94	0.84	0.60	0.92
	Vowel2 local intensity	25	0.84	0.67	0.93	0.81	0.57	0.93
Vowels	V1 F1	25	0.25	−0.16	0.59	0.25	−0.31	0.67
	V1 F2	25	0.65	0.34	0.83	0.65	0.10	0.91
	V1 F3	25	0.84	0.67	0.93	0.78	0.62	0.88
	V1 aperiodicity	25	0.60	0.27	0.80	0.56	0.21	0.78
	V1 centroid	25	0.86	0.69	0.94	0.85	0.60	0.93
	V1 intensity	25	0.79	0.57	0.90	0.73	0.37	0.86
	V2 F1	25	0.80	0.59	0.91	0.73	0.31	0.88
	V2 F2	25	0.82	0.64	0.92	0.79	0.53	0.94
	V2 aperiodicity	25	0.28	−0.13	0.60	0.24	−0.04	0.50
	V2 centroid	25	0.74	0.48	0.88	0.63	0.22	0.84
	V2 intensity	25	0.61	0.28	0.81	0.49	0.18	0.75
Vowel Geometry	Standardized inter-vowel distance	25	0.73	0.47	0.87	0.72	0.19	0.95
	Vowel distance F1–F2	25	0.50	0.13	0.75	0.47	0.14	0.84
	ΔF1 (V2–V1)	25	0.71	0.44	0.86	0.68	0.06	0.89
	ΔF2 (V2–V1)	25	0.60	0.26	0.80	0.54	0.21	0.78

Note: The /s/ attack 10–90% descriptor was evaluable in 24 pairs, T−3 dB post-vowel decay in 22 pairs, and T−6 dB post-vowel decay in 21 pairs because insufficient segment energy prevented calculation in the remaining recordings. No new Core, Candidate, or Secondary tiers were assigned in this within-sample sensitivity analysis.

Supplementary Methods

Supplementary Technical Validation

The design and main results of the technical validation are reported in Sections 2.4 ("Validation of the External Recording Pathway") and 3.1 ("Measurement-System Validation") of the main text. This section adds only procedural details not given there and presents the corresponding tables and figures: the overall workflow is shown in Figure SB1, and the components and pre-specified criteria are listed in Table SA1.

Primary electrical loopback. Pink and white noise (60 s, 24-bit, peak −3.00 dBFS) were played from the headphone output of one computer and recorded in Praat on a second computer. Three 30-s recordings per stimulus were aligned to the source by cross-correlation. Because no calibrated input could be injected at the FreeFit monitoring stage, the test covered the pathway from the cable onwards. A source–recording correlation of 0.95–0.98 and a clock drift of approximately 13 ppm confirmed an analogue signal path, and a numerically applied first-order 2000-Hz low-pass filter was recovered to within a few tenths of a decibel. The maximum deviation of the three-trial median was 0.037 dB (1600 Hz, both stimuli), with a maximum across-trial range of 0.036 dB.

Primary acoustic validation. The ISTS lasted 14 s and was presented at 65 dB SPL; OTOsuite spectra were obtained with the Freestyle function. Within-path repeatability is detailed in Table SA2, the underlying spectra in Tables SA3 and SA4, and cross-path agreement in Table SA5 and Figure SB2. Relative to the OTOsuite display, the external recording showed more energy at 250–500 Hz and less at 4–6 kHz.

Secondary stereo bridge. Band-by-band electrical transfer is reported in Table SA6 and Figure SB3, and bilateral acoustic repeatability and cross-path agreement in Table SA7. The resulting interpretation of all tests is summarised in Table SA8.

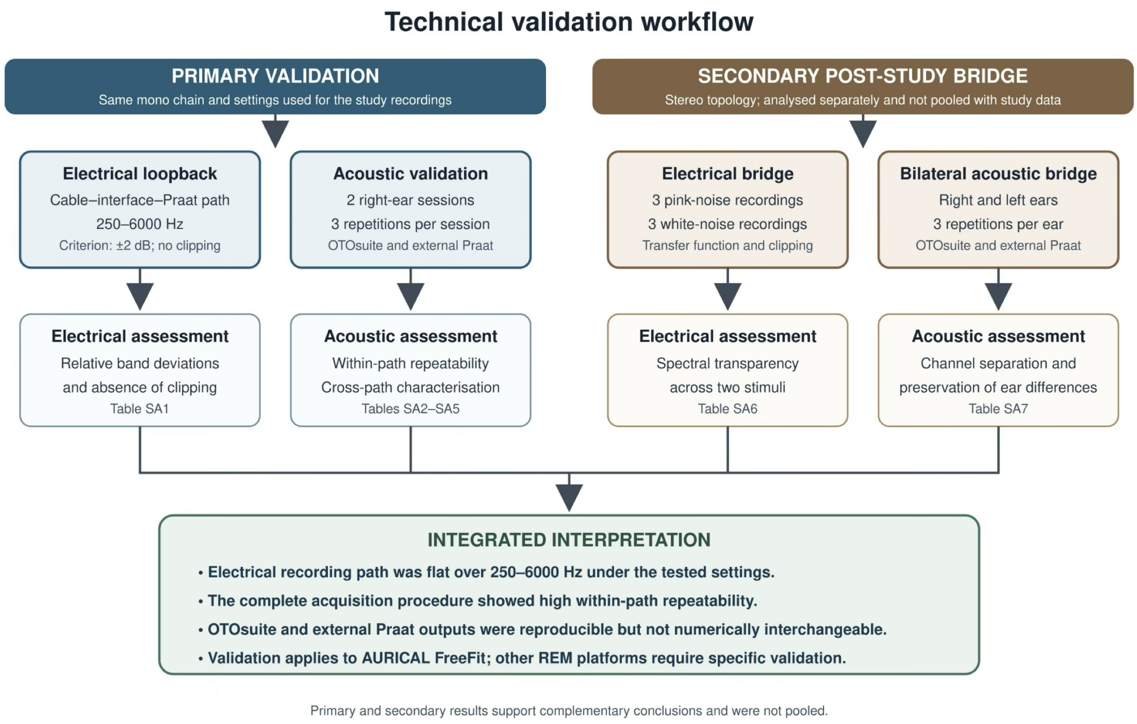

Figure SB1. Technical validation workflow used to evaluate the external SpettroREM acquisition pathway. The primary validation reproduced the mono cable and acquisition settings used for the study recordings and comprised electrical loopback, two right-ear acoustic sessions with three repetitions per session, within-path repeatability, and cross-path characterisation against OTOsuite. The secondary post-study stereo bridge comprised independent pink- and white-noise electrical recordings and bilateral acoustic repetitions to test cable topology, channel separation, and preservation of ear-related differences. Primary and secondary data were analysed separately and were not pooled. The integrated interpretation is restricted to the tested AURICAL FreeFit configuration; transfer to other REM platforms requires platform-specific validation or harmonisation.

Table SA1. Validation components and pre-specified interpretation.

**Component**	**Purpose**	**Frequency range / repetitions**	**Criterion or interpretation**
Primary electrical loopback (mono)	Test cable–interface–Praat frequency response	250–6000 Hz	All relative band deviations within ±2 dB; no clipping
Primary acoustic repeatability	Test stability of the complete measurement procedure	2 sessions × 3 right-ear repetitions	High within-path correlations; small across-trial band range
Primary cross-path comparison	Characterise, not assume, OTOsuite/external agreement	9 one-third-octave bands	Report r, MAE, RMSE, and maximum difference; no requirement of numerical equivalence
Secondary stereo electrical bridge	Test cable topology with two independent stimuli	3 pink + 3 white recordings	Near-flat response supports electrical transparency
Secondary bilateral acoustic bridge	Test channel separation and preservation of ear differences	3 repetitions per ear	Repeatability and concordance of interaural-difference pattern

Table SA2. Primary mono-cable acoustic quality control and repeatability.

Session	Path	Pairwise r, range	Maximum across-trial band range (dB)	Probe calibration / note
1	OTOsuite	0.958–0.967	3.82	Right-ear calibration: 69, 70, and 68 dB
1	External Praat	0.985–0.996	3.85	Same fixed mono chain
2	OTOsuite	0.969–0.985	2.11	Successful probe calibration
2	External Praat	0.976–0.990	3.66	Same fixed mono chain

Table SA3. OTOsuite one-third-octave values from the primary right-ear acoustic validation (dB SPL).

Session	Trial	250	500	750	1000	1500	2000	3000	4000	6000
1	1	58	58	56	47	54	57	63	55	46
1	2	58	59	56	49	53	54	63	54	47
1	3	58	59	56	50	53	56	66	56	43
2	1	56	59	57	52	54	58	61	55	46
2	2	55	60	57	51	53	56	62	53	44
2	3	57	59	56	53	54	57	62	56	45

Table SA4. Mean-normalised external Praat spectra from the primary right-ear acoustic validation (dB).

Session	Trial	250	500	750	1000	1500	2000	3000	4000	6000
1	1	6.92	9.68	4.47	-6.07	0.64	2.60	5.53	-5.06	-18.70
1	2	6.86	9.92	4.17	-5.43	-0.27	1.20	5.57	-3.87	-18.16
1	3	6.45	9.59	3.38	-5.21	-0.74	0.97	8.37	-3.31	-19.49
2	1	8.11	9.86	4.92	-2.14	-0.05	3.45	2.09	-5.45	-20.80
2	2	5.51	10.04	5.84	-1.88	0.16	1.18	5.60	-4.14	-22.30
2	3	7.34	9.04	3.38	-1.09	-1.26	0.78	5.75	-3.05	-20.89

Table SA5. Primary cross-path spectral-shape agreement.

Session	Trial	Pearson r	MAE (dB)	RMSE (dB)	Maximum |difference| (dB)
1	1	0.848	3.9	4.77	9.81
1	2	0.869	3.58	4.53	10.38
1	3	0.913	3.06	3.82	7.27
2	1	0.847	4.48	5.56	11.47
2	2	0.91	3.65	4.85	11.74
2	3	0.912	3.47	4.67	10.44

Table SA6. Secondary stereo electrical bridge: mean relative transfer by band (dB).

Centre frequency (Hz)	Pink noise, mean of 3	White noise, mean of 3
250	-0.020	-0.085
500	-0.030	0.071
750	0.050	0.008
1000	-0.060	-0.016
1500	0.050	-0.019
2000	0.040	0.013
3000	-0.040	-0.006
4000	0.000	0.025
6000	0.020	0.010

Across individual recordings, the maximum absolute band deviation was 0.08 dB for pink noise and 0.091 dB for white noise. No clipping was observed. The mean absolute difference between the pink- and white-noise transfer estimates was approximately 0.045 dB.

Table SA7. Secondary stereo acoustic bridge: repeatability and cross-path agreement.

Ear / path	Within-path pairwise r	Cross-path r	Cross-path MAE (dB)	Maximum |difference| (dB)
Right, external Praat	0.996–0.998	0.790–0.850	4.67–4.93	10.18–12.91
Right, OTOsuite	0.977–0.993	—	—	—
Left, external Praat	0.996–0.998	0.882–0.901	4.01–4.24	9.43–11.37
Left, OTOsuite	0.990–0.995	—	—	—

Table SA8. Interpretation matrix.

Question	Finding	Supported conclusion
Is the electrical recording path spectrally flat?	Primary loopback passed ±2 dB; secondary bridge maximum = 0.091 dB	Yes, over 250–6000 Hz under the tested settings.
Is the complete procedure repeatable?	Within-path r up to 0.996 (primary) and 0.998 (bridge)	Yes; random acquisition instability is small relative to the spectral pattern.
Are external and OTOsuite spectra numerically interchangeable?	MAE 3.06–4.48 dB; maximum 11.74 dB in primary tests	No. They must be treated as distinct processing outputs.
Was the mono cable the principal cause of the discrepancy?	The discrepancy persisted with a stereo splitter	Not supported by the bridge experiment.
Does the external pathway preserve ear-related differences?	Interaural-difference r = 0.850; MAE = 0.704 dB	Yes, within the tested bilateral bridge measurement.
Can results be transferred automatically to other REM systems?	Only AURICAL FreeFit/OTOsuite was validated	No; platform-specific validation or harmonisation is required.

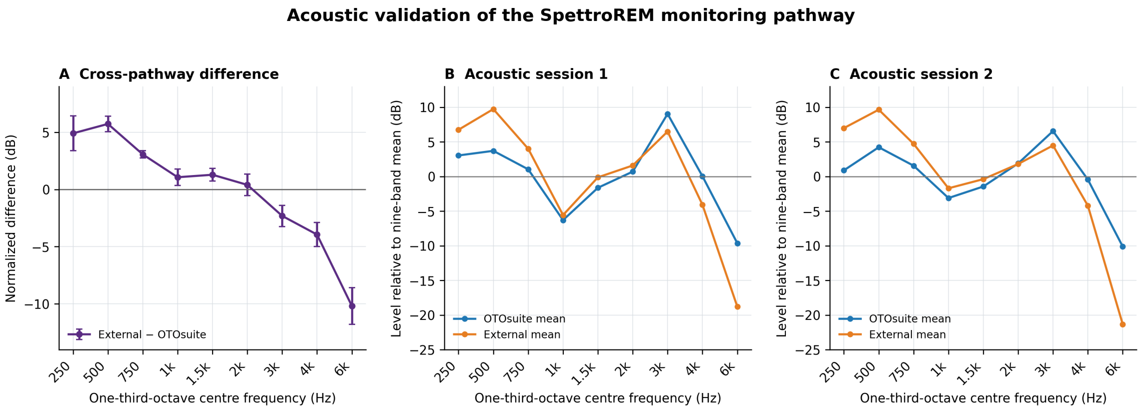

Figure SB2. Primary mono-cable acoustic validation of the external SpettroREM acquisition pathway. Panel A shows the mean normalized difference between the simultaneous external recording and the OTOsuite spectrum (external minus OTOsuite); error bars represent the standard deviation across the six paired acoustic recordings. Panels B and C show the mean-normalized one-third-octave spectra obtained from OTOsuite and the simultaneous external Praat recordings during acoustic sessions 1 and 2, respectively. Normalization was performed by subtracting the mean across the nine frequency bands from each spectrum. The cross-path comparison was descriptive and was not evaluated against the prespecified ±2-dB electrical-loopback criterion. The results demonstrate reproducible within-path spectral shape while confirming that the OTOsuite display and the monitor-derived external recording should not be treated as numerically interchangeable outputs.

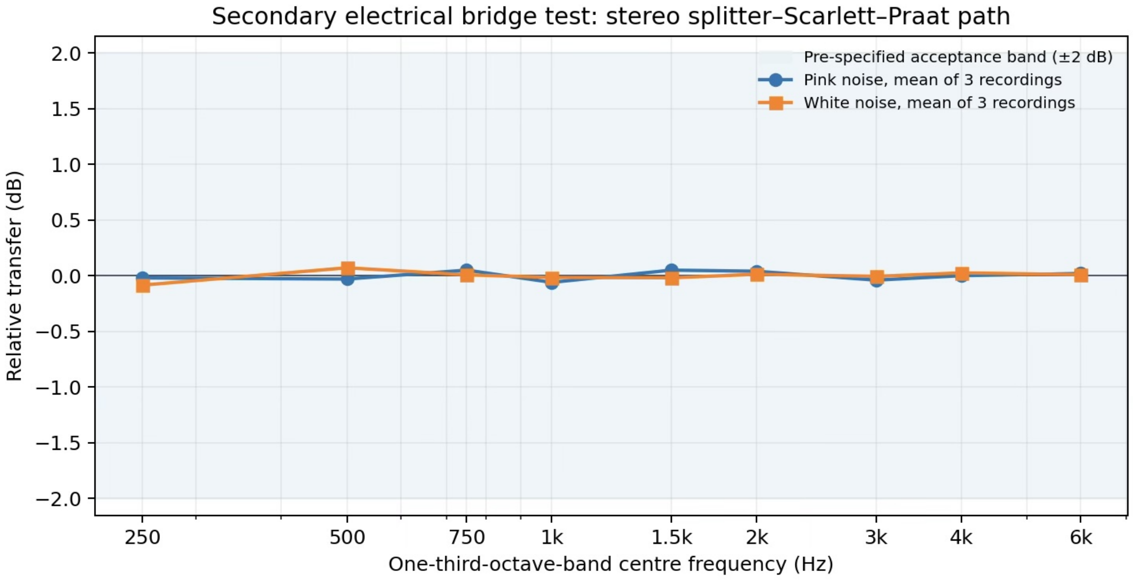

Figure SB3. Mean relative transfer functions obtained in the post-study stereo bridge experiment using three pink-noise and three white-noise recordings. Spectra were normalised by removal of the overall level offset. All individual deviations were substantially smaller than the pre-specified ±2-dB acceptance band (maximum observed deviation, 0.091 dB).

Reference

Koo, T.K.; Li, M.Y. A guideline of selecting and reporting intraclass correlation coefficients for reliability research. *J. Chiropr. Med.* **2016**, *15*, 155–163. https://doi.org/10.1016/j.jcm.2016.02.012.
